# Weaning transition, but not the administration of probiotic candidate *Kazachstania slooffiae*, shaped the gastrointestinal bacterial and fungal communities in nursery piglets

**DOI:** 10.3389/fvets.2023.1303984

**Published:** 2024-01-11

**Authors:** KaLynn Harlow, Katie Lynn Summers, William T. Oliver, James E. Wells, Matthew Crouse, Bryan W. Neville, Lea A. Rempel, Israel Rivera, Timothy G. Ramsay, Cary Pirone Davies

**Affiliations:** ^1^Oak Ridge Institute for Science and Education, Agricultural Research Service Participation Program, Oak Ridge, TN, United States; ^2^Animal Biosciences and Biotechnology Laboratory, Beltsville Agricultural Research Center, Agricultural Research Service, United States Department of Agriculture, Beltsville, MD, United States; ^3^Meat Animal Research Center, Agricultural Research Service, United States Department of Agriculture, Clay Center, NE, United States

**Keywords:** fungi, microbiome, mycobiome, bacteriome, pig, Kazachstania slooffiae

## Abstract

As in-feed antibiotics are phased out of swine production, producers are seeking alternatives to facilitate improvements in growth typically seen from this previously common feed additive. *Kazachstania slooffiae* is a prominent commensal fungus in the swine gut that peaks in relative abundance shortly after weaning and has beneficial interactions with other bacteriome members important for piglet health. In this study, piglets were supplemented with *K. slooffiae* to characterize responses in piglet health as well as fungal and bacterial components of the microbiome both spatially (along the entire gastrointestinal tract and feces) and temporally (before, during, and after weaning). Litters were assigned to one of four treatments: no *K. slooffiae* (CONT); one dose of *K. slooffiae* 7 days before weaning (day 14; PRE); one dose of *K. slooffiae* at weaning (day 21; POST); or one dose of *K. slooffiae* 7 days before weaning and one dose at weaning (PREPOST). The bacteriome and mycobiome were analyzed from fecal samples collected from all piglets at day 14, day 21, and day 49, and from organ samples along the gastrointestinal (GI) tract at day 21 and day 49. Blood samples were taken at day 14 and day 49 for cytokine analysis, and fecal samples were assayed for antimicrobial resistance. While some regional shifts were seen in response to *K. slooffiae* administration in the mycobiome of the GI tract, no remarkable changes in weight gain or health of the animals were observed, and changes were more likely due to sow and the environment. Ultimately, the combined microbiome changed most considerably following the transition from suckling to nursery diets. This work describes the mycobiome along the piglet GI tract through the weaning transition for the first time. Based on these findings, *K. slooffiae* administered at this concentration may not be an effective tool to hasten colonization of *K. slooffiae* in the piglet GI tract around the weaning transition nor support piglet growth, microbial gut health, or immunity. However, diet and environment greatly influence microbial community development.

## Introduction

The weaning transition in swine is a critical period whereby significant changes in diet and environment result in increased stress, enhanced predisposition to opportunistic diseases, and expansive changes in the microbiome (1–3). In addition, movement to nursery pens results in piglets from multiple litters having to intermingle and establish new social hierarchies. The fact that these events happen concurrently amplifies the perceived stress. Increased stress increases potential damage to the gastrointestinal (GI) tract and enhances susceptibility to disease from opportunistic pathogens, including resident bacteria and fungi in the digesta and mucosal lining of the GI tract. Until recently, in-feed antibiotics were utilized to combat opportunistic diseases and enhance weight gain in piglets, but with recent changes on use of antibiotics, creative approaches are needed to discover alternative growth promotants and methods to support gut health and immunity. Probiotics have been investigated as an alternative to support the total microbiome through stressful transitions that can result in periods of dysbiosis, and this is accomplished with high consumer approval from the general public (4).

Microbial colonization of the GI tract starts at birth. The fungal community within the microbiome (mycobiome) is a ubiquitous, but often neglected, component of the microbiome and its role in swine health remains to be understood. Despite being numerically inferior to the bacterial population within the microbiome (bacteriome) (5, 6), the mycobiome has been shown to alter host immunity in healthy animals and alter the severity of some diseases. Opportunistic fungal pathogens have been found to colonize the gut and induce altered immune responses that can result in disease or altered disease states (7–9). Further, some fungi commonly found in the gut can alter the taxonomy and function of the bacteriome, resulting in altered health (10–12). Previously, characterization of the fecal microbiome as a representation of the gastrointestinal tract was important due to its noninvasive nature. However, bacterial and fungal colonization differs markedly between regions of the GI tract (13–16). Therefore, a complete observation of discrete spatial microbiomes in conjunction with the fecal microbiome, which is representative of the transfer of content between these regions, can reveal more information about the potential for disease state in piglets during this critical transition.

In previous studies, we have demonstrated the dominance of a single fungal species, *Kazachstania slooffiae*, in the GI tract of pigs from weaning and through their lifespan (13–16). *K. slooffiae* has also been found to have beneficial interactions with bacteriome members, such as *Lactobacillus*, known for promoting health in the pig gut (13). Further, negative inferred interactions were found for *K. slooffiae* and potential pathogens such as *Aspergillus* (13). Based on its dominance in the pig GI tract (17), its lack of known ability to cause disease, and preliminary studies suggesting its beneficial role in the gut (18, 19), we hypothesized that increasing the abundance of *K. slooffiae* through supplementation before the weaning transition would quicken its establishment in the GI tract mycobiome and help alleviate detrimental effects seen during this time. We performed a feeding trial in piglets throughout the weaning transition to document the effects of supplemental *K. slooffiae* on piglet health, weight gain, cytokine response, and changes to antimicrobial resistance (AMR), as well as characterize the myco- and bacteriome both spatially (along the entire GI tract and feces) and temporally (before, during, and after weaning).

## Materials and methods

### Animals

This animal study was reviewed and approved by the USDA-ARS Institutional Animal Care and Use Committee of the Meat Animal Research Center (Project # 133.0). In total, 363 piglets (from established USMARC Landrace-Duroc-Yorkshire dams bred to industry relevant Yorkshire sires) from 32 litters were assessed from farrowing through day 49 of life and were weaned at day 21. Litters were used from two different, subsequent batch farrowings, during which sows and piglets were maintained in adjacent rooms. Sows and litters were housed in individual farrowing crates for the entirety of lactation, and litters were standardized to 10 to 11 piglets per litter with cross-fostering completed within the first week of life. Piglets were not provided milk replacer/supplement or creep feed at any point throughout the experiment and were evaluated to be healthy. No antibiotics, antifungals, or supplementary additives were administered to the piglets at any time during the experiment. At weaning, piglets were moved by litter to nursery pens and given a commercially-available transition starter diet for 2 days followed by two different phase diets (Phase 2: two days after weaning through 14 days after weaning; Phase 3: 15 days after weaning through 28 days after weaning), all of which were formulated to meet or exceed the National Research Council estimate of nutrient requirements (20).

### Weight gain calculations

Average daily gain was calculated for each growth interval by subtracting the start weight from the end weight of each individual piglet and subsequently dividing by the number of days in the period. Statistical analysis was completed using the standard least squares fit model in JMP^®^ (Version 15.2.0, SAS Institute Inc., Cary, NC, 1998–2023). Treatment, age, and treatment by age interactions were considered as fixed effects, and pairwise comparisons were analyzed using Student’s *t*-tests.

### Fungal culturing and treatment

*K. slooffiae* was originally isolated from fecal samples collected from post-weaning piglets (~24 days of age). Briefly, samples were weighed, homogenized in 1X phosphate buffered saline (PBS) in a biological safety cabinet and plated on Yeast Potato Dextrose (YPD) agar supplemented with 0.1 mg/mL cefoperazone (“Cef,” to prevent bacterial growth) at 37° C. Colonies that grew were Sanger sequenced using primers specific to *K. slooffiae* (21). One confirmed colony was grown in YPD broth + Cef and subsequently subjected to whole-genome sequencing using the PacBio Sequel II 18 M SMRT Cell platform (22). Further *K. slooffiae* culture was grown and cryopreserved at −80° C in YPD broth supplemented with 10% per volume 100% sterile glycerol (Fisher Scientific, Hampton, NH) for future use. This isolated strain has previously been shown to have beneficial interactions *in vitro* with *Lactobacillus acidophilus* (22). *K. slooffiae* was isolated from a single colony and grown in 5 mL of YPD supplemented with 0.1 mg/mL Cef at 37° C and shaking at 200 rpm. After 24 h of growth, the 5 mL culture was added to 20 mL of YPD + Cef and cultured for another day. In this way, the fungal culture was grown to sufficient levels to feed to piglets in the experiment. On the day of experiments, cultures were spun down at 168 × g for 5 min. Cultures were washed in sterile 1X PBS and spun down again prior to resuspension in sterile 1X PBS. Final culture was replica plated on YPD agarose + Cef to confirm the dose fed to pigs. An approximate dosage of 2 × 10^6^ CFU/pig was given for each oral treatment.

Litters containing both gilts and barrows were randomly assigned to one of four treatment groups, which resulted in eight litters per treatment. The CONT group received an oral gavage of sterile 1X PBS (*n* = 90 piglets). The PRE treatment group received a single oral gavage of *K. slooffiae* at 1 week pre-weaning (*n* = 89 piglets). The POST treatment group received a single oral gavage of *K. slooffiae* at the time of weaning (*n* = 92 piglets). The PREPOST treatment group received an oral gavage of *K. slooffiae* at 1 week pre-weaning and at the time of weaning (*n* = 92 piglets).

### Sample collection

Each piglet was weighed throughout the experiment and fecal samples were collected on days 14, 21, and 49. Piglets were followed until day 49 of age (end of nursery). Fecal samples were collected directly from the rectum of the piglet into a sterile basin and then transferred to a 2.0 mL tube and immediately frozen in liquid nitrogen until transport to the laboratory. Blood samples were collected on days 14 and 49 and placed on ice until transport to the laboratory where they were spun for 25 min at 1000 × g at 4°C. Each subsequent serum sample was saved as multiple aliquots and frozen at −80°C until later analyses. On day 21 (weaning) and 49 (end of nursery), one representative barrow (median weight) was taken from each litter and euthanized for further study (*n* = 8 barrows per timepoint per treatment). Organ samples were taken of the stomach, duodenum, jejunum, cecum, and colon. In addition, fecal samples were taken directly from the rectum. Each sample of the GI tract was briefly rinsed in sterile 1X PBS to remove residual digesta. Feces or GI tract samples were placed into individual, sterile, twist top tubes, flash frozen, and stored in liquid nitrogen until all samples were placed in a −80°C freezer at the end of sample collection that day. On days where treatments overlapped with sample collection, samples were collected before treatment.

### DNA extraction and sequencing

DNA was isolated from 0.25 g of feces or GI organ tissue samples using the MagAttract Power Microbiome Kit (Qiagen, Hilden, Germany) by the Microbial Systems Molecular Biology Laboratory at the University of Michigan. Cells were lysed to isolate DNA using mechanical bead beating for 20 total minutes with 20 frequency/s and extracted using magnetic bead technology according to the Qiagen protocol. While the bacteriome and mycobiome does not reside in the organ tissue itself, and the residual digesta was rinsed during sample collection, this extraction and subsequent amplification was used to target DNA from the resident microbes in the mucosa adhering to the organ samples collected. “Organ region” will be used to refer to the microbial community present in the mucosa in that region of the GI tract.

### Microbiome processing and sequencing (16S – bacteriome)

The V4 region of the 16S rRNA-encoding gene was amplified from extracted DNA using the barcoded dual-index primers developed previously (23). Samples were sequenced on the Illumina MiSeq sequencing platform at the University of Michigan, generating 250 bp paired-end reads. Sequence results are publicly accessible at NCBI BioProject ID PRJNA1020867.

### Microbiome processing and sequencing (ITS – mycobiome)

The ITS region was sequenced utilizing primers ITS3 (5′ GCATCGATGAAGAACGCAGC 3′) and ITS4 (5′ TCCTCCGCTTATTGATATGC 3′) with the Illumina adaptor sequence added to the 5′ end. Samples were sequenced on the Illumina MiSeq sequencing platform at the Animal Biosciences and Biotechnology Laboratory at the Beltsville Agricultural Research Center in Beltsville, MD, generating 300 bp paired-end reads. Sequence results are publicly accessible at NCBI BioProject ID PRJNA1020867.

### Microbiome analyses (16S – bacteriome)

Quality filtering, pairing, denoising, amplicon sequence variant (ASV) determination, and chimera removal were conducted with the DADA2 plugin (24) in QIIME2 v. 2019.7 (25). For quality trimming, paired-end sequences were truncated to 240 and 160 bp for forward and reverse reads, respectively. Taxonomic classification of the ASVs was performed using the pretrained 16S 515F/806R from the Silva 138 database (26). ASVs identified as Archaea, chloroplast, mitochondria, or unassigned were removed from further analysis. Rarefaction curves were plotted in QIIME2, and a threshold of 5,000 reads was selected as the minimum sequencing depth for each sample. The average number of reads per sample for 16S data were 25,327. Samples <5,000 sequences were removed from downstream analyses (*n* = 412 fecal samples, *n* = 197 GI mucosa samples).

### Microbiome analyses (ITS – mycobiome)

Due to the biologically relevant length variation found in fungal ITS sequences, Trimmomatic v 0.39 (27) and the sliding window option were used to trim individual sequences where the average quality score was <15 across 4 base pairs. Reads were then imported into QIIME2 version 2021.11 for further analysis. Cutadapt was used to remove forward and reverse primers from paired reads (28). Amplicon sequence variants (ASVs) were identified using the DADA2 plug-in. The QIIME2 formatted UNITE fungal full-length ITS database version 8.3 (clustered at 99%) (29) was downloaded and imported into QIIME2. Taxonomic classifications were assigned to ASVs using a naïve Bayes classifier trained on the UNITE database. Rarefaction curves were plotted in QIIME2, and a threshold of 8,000 reads was selected as the minimum sequencing depth for each sample. The average number of reads per sample for ITS data were 64,681. Samples <8,000 sequences were removed from downstream analyses (*n* = 375 fecal samples, *n* = 281 GI organ samples; numbers include some duplicate samples).

### Characterization of the bacteriome and mycobiome

Statistical calculations were performed in R (30), and feature tables were exported from QIIME2 and imported into R using the package qiime2R (31). To estimate alpha diversity, Shannon diversity was calculated on piglet fecal and GI mucosa samples using the vegan package (32). To determine significant trends in alpha diversity, mixed-effects linear models were calculated using the lmer function in the lme4 package (33), with both sow and piglet nested within sow as the random variables and age (day 14, day 21, day 49 for fecal samples; day 21 and day 49 for GI organ region samples), treatment (CONT, PRE, POST, and PREPOST), farrowing group (1 or 2), sex (only for fecal samples), or organ (only for GI organ region samples) as fixed-effect variables. Posthoc pairwise testing was performed using the emmeans function within the emmeans package (34). To visualize beta-diversity, principal components analysis (PCA) of piglet communities were conducted using the rda function in the vegan package (32) on either hellinger transformed (16S) or log-transformed (ITS) bacterial and fungal sequence counts using Bray–Curtis dissimilarity distances. To reduce potential ASV artifacts, ASVs present in <2.0% of samples were removed prior to analysis. Values for PC1 and PC2 were used as outcome variables to model the effects of fixed and random variables (as above) on beta-diversity. This method was selected instead of typical PERMANOVA analysis as PERMANOVA requires balanced input data, and roughly one third of our data would have been lost in this balancing process. Due to R software limitations, this analysis was performed using PROC GLM in SAS (Version 9.4 M8; Cary, NC) with LSMEANS to estimate Posthoc pairwise comparisons. PROC VARCOMP was used to estimate the proportion of total variance attributable to random effects. For all alpha and beta diversity models, SAS function GLMSELECT was utilized to perform stepwise regression and reduce model complexity. All data in the manuscript are plotted with ggplot2 (35).

### Pathogenic bacteria and antimicrobial resistance assays

Rectal swabs were collected from all piglets at end of farrowing and nursery phases for molecular determination of *Campylobacter*, *Salmonella*, and shigatoxigenic *Escherichia coli (E. coli)* as described previously (36). Specifically, presence of *Campylobacter coli (C. coli)*, thermophilic species of *Campylobacter* (abbreviated here as C. therm), *Salmonella*-specific *invA*, *hlyA* (*E. coli* hemolysin), *eaeA* (*E. coli* attaching and effacing gene), *stx1* and *stx2* (genes in *E. coli* that produce Shiga toxin) were determined. Briefly, the rectum of each piglet was swabbed with a sterile cotton swab and each swab was placed into a 15 mL conical tube with 2 mL of tryptic soy broth, capped and vigorously vortexed. A subsample was removed and stored frozen, and the remainder was enriched for 6 h at 37°C and then frozen for *Salmonella* and *E. coli* detections. Presence in pre-enriched subsamples was assayed by PCR for thermophilic *Campylobacter* (37) and for specific *Campylobacter* species (38). Presence of *Salmonella* in enriched samples was determined using PCR for *invA* gene (39). Potential colonization for shigatoxigenic *E. coli* were determined using multiplex PCR for virulence genes (40). Percent prevalence of genes for targeted pathogen or virulence genes are reported for each litter housed in separate pens at weaning (*n* = 32 litters) and in the nursery (*n* = 32 litters). Additionally, cecal and fecal samples were collected from euthanized piglets at end of farrowing and nursery phases and 1 g of content was diluted 10-fold in tryptic soy broth. Samples were then cultured on plates to discriminate antibiotic-resistant bacteria. A blinded observer enumerated samples for total *E. coli*, total tetracycline-resistant (TETr) *E. coli*, total trimethoprim-sulfamethoxazole-resistant (COTr) *E. coli*, and total cefotaxime-resistant (CTXr) *E. coli* similar as reported previously (41). Statistical analysis was completed using the general linear model in JMP^®^ (Version 15.2.0, SAS Institute Inc., Cary, NC, 1998–2023). Treatment, age, and treatment by age interactions were considered as fixed effects, and pairwise comparisons were analyzed using Student’s *t*-tests. All values reported are least squares means ± standard error (SEM).

### Immunological responses

Cytokines were measured from serum samples collected at day 14 and day 49. Cytokines measured include: IFNγ, IL-1α, IL-1β, IL-1Ra, IL-2, IL-4, IL-6, IL-8, IL-10, IL-12, IL-18, and TNFα (MAGPIX, Luminex, Austin, TX; kit from EMD Millipore, Billerica, MA). Statistical analysis was completed using the standard least squares fit model in JMP^®^ (Version 15.2.0, SAS Institute Inc., Cary, NC, 1998–2023). Treatment, age, and treatment by age interactions were considered as fixed effects, and pairwise comparisons were analyzed using Student’s *t*-tests. All values are reported as least squares means ± standard error (SEM).

## Results

### Growth rate

**Figure 1:**
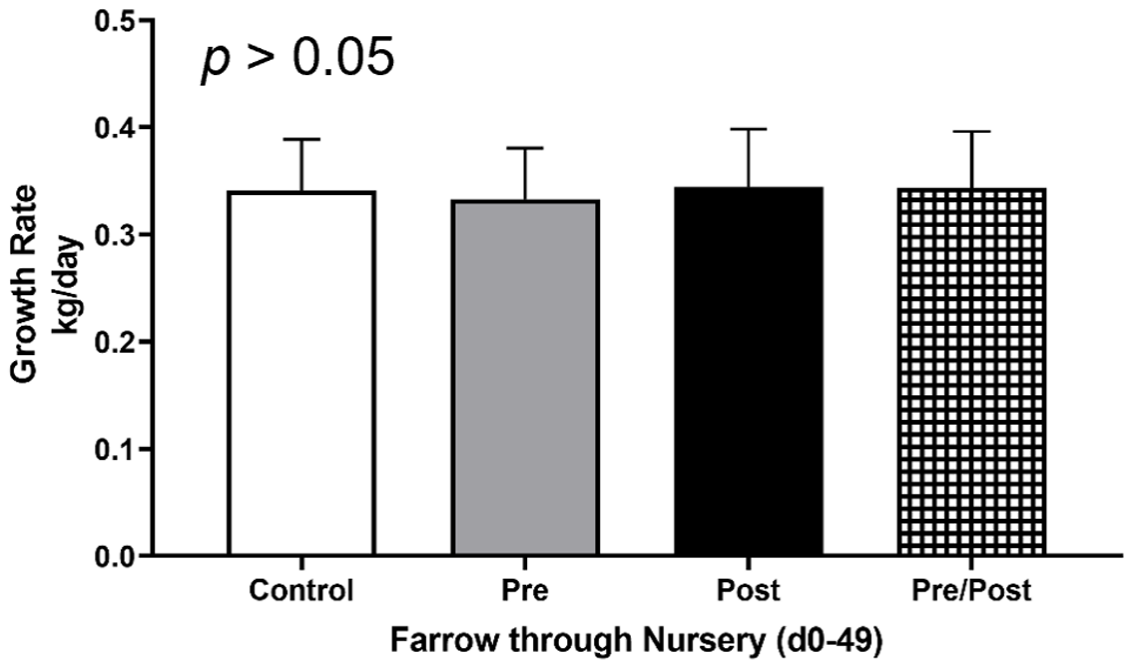
Growth rates of piglets from farrow (day 0) to end of nursery phase (day 49). Piglets were weighed throughout the experiment to assess growth rate of kg/day. No differences were found as an effect of treatment. (CONT: *n* = 90 piglets; PRE: *n* = 89 piglets; POST: *n* = 92 piglets; PREPOST *n* = 92 piglets).

To determine if treatments influenced average daily gain (ADG) of piglets, weights were taken at multiple time points throughout the trial (Figure 1). No differences were seen between treatment groups for ADG from birth until end of treatment at day 49 (*p* > 0.05). Additionally, no differences were seen between treatments for rate of growth during different time frames (i.e., birth to day 14, day 14 to day 49, or day 21 to day 49; *p* > 0.05).

### Composition and diversity of the bacteriome along the GI tract and in fecal samples

**Figure 2:**
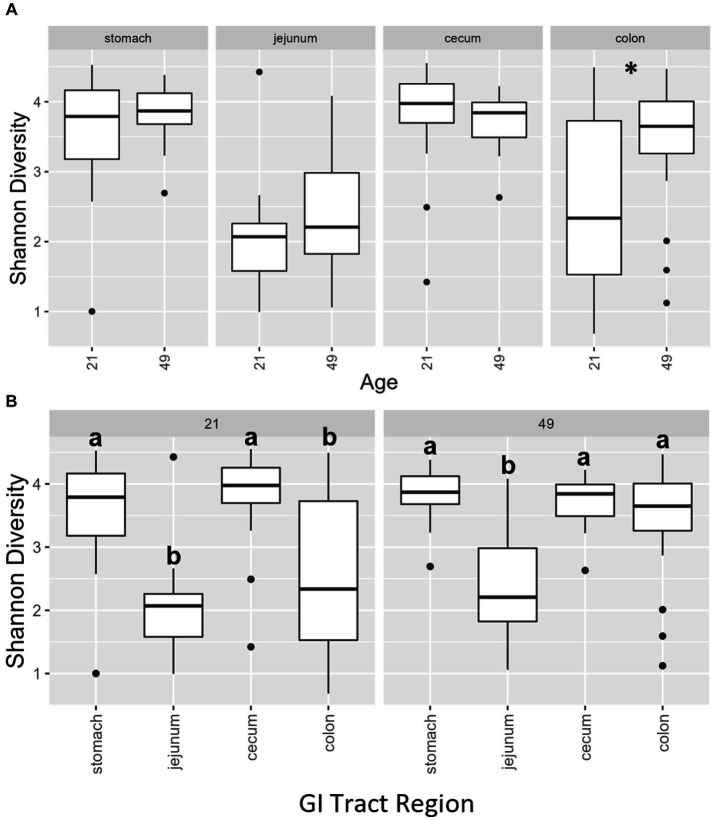
**(A)** Shannon diversity values of the bacteriome in GI organ regions (age by organ). Significant differences between ages only occur in the colon. **(B)** Shannon diversity values of the bacteriome in organ samples (organ by age). Alpha diversity in the colon was greater in older animals relative to younger animals. *, significantly different between age groups within organ. Differing lowercase letters indicate significant differences between organs but within age. Dots represent outliers. (*n* = 8 barrows per timepoint per treatment).

**Figure 3:**
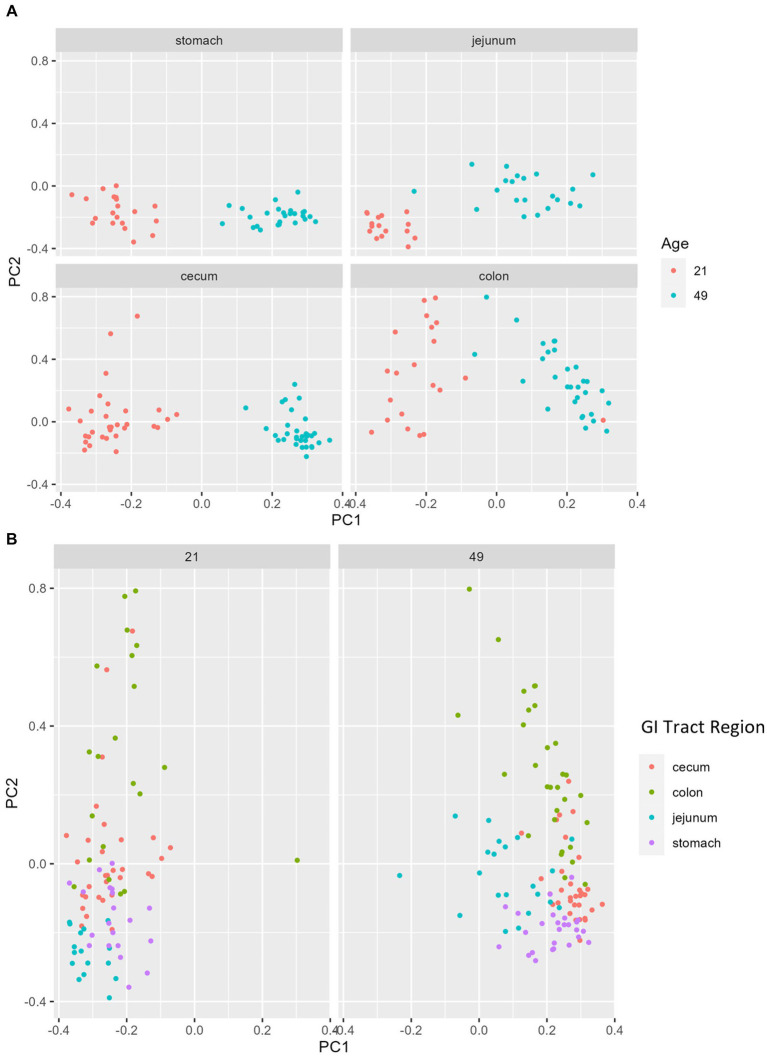
PCA plots of Bray–Curtis dissimilarity of the bacteriome in GI organ regions by age. **(A)** Within organ region, communities were distinguishable by age (*p* < 0.05). **(B)** All organs regions were significantly different than each other (*p* < 0.05) except the stomach and jejunum at day 21, and the cecum and stomach at day 49. (*n* = 8 barrows per timepoint per treatment).

Multiple regions along the GI tract as well as fecal samples were analyzed for bacterial composition and diversity to make both temporal and spatial comparisons and to assess the impact of treatment on microbiome communities. Farrowing group (1 and 2) and sex of the piglets (gilts and barrows) were also taken into consideration when determining model selection. For 16S organ samples, Shannon values were compared across age, GI organ region, farrowing group, and treatment. Notably, treatment was excluded from the model following the GLMSELECT procedure. For a complete list of variables included in linear mixed models after model selection for all analyses, see Table 1. In 16S data, duodenal samples were removed from analysis due to low read counts, and jejunum sampling was incomplete as one treatment group was missing (PRE). Shannon values differed significantly across age, GI organ region, and farrowing group (*p* < 0.05). Both GI organ region and farrowing group had interactions with age (*p* < 0.05; see Supplementary Table S1 for complete overview of all interactions for this and subsequent analyses). Shannon diversity was greater in older animals than younger ones in the colon (*p* < 0.0001), and marginally significant in the jejunum in samples that could be analyzed (*p* = 0.048), but this did not change significantly over time in the cecum nor stomach (Figure 2A). Differences in Shannon values can also be seen between some GI organ regions at each timepoint (Figure 2B). At day 21, Shannon values were higher in the stomach and cecum than in the jejunum and colon, while at day 49, Shannon values increased in the cecum to reach levels observed in the colon and stomach, while remaining low in only the jejunum. Finally, there was an impact of farrowing on diversity measures, but only at day 21, where Shannon values were higher in farrowing group 1 than in farrowing group 2 (*p* = 0.0030). In GI tract samples, age and organ region were significant in models for beta-diversity, as were interactions between age and organ region, and organ and farrowing group (*p* < 0.05). There were no significant differences in overall microbiome community composition across treatments (*p* > 0.05). Within each region of the GI tract, communities were distinct at day 21 and day 49 (Figure 3A). At each timepoint, regional GI tract communities differed from one another along the GI tract (*p* < 0.0001; Figure 3B). When taking both PC1 and PC2 into consideration, which accounted for 16% (PC1) and 8% (PC2) of the diversity in this population, all GI organ regions differed from all others (*p* < 0.05) except the jejunum was not significantly different than the stomach at day 21 (*p* > 0.05). At day 49, all GI organ regions were significantly different than all others (*p* < 0.05) except for the cecum and stomach (*p* = 0.056) and colon and stomach (*p* = 0.26); however, these groups are visually distinguishable in plots showing PC1 and PC2.

**Table 1:** Table of complete list of variables included in linear mixed models after model selection in GLM SELECT.

Analysis	Final model	
16S organ region – alpha diversity	Shannon = age organregion farrow age*organregion age*farrow (1|sow/pig)	
16S organ region – beta diversity	pc1 pc2 = age organregion farrow treatment organregion*age organregion*farrow	
16S feces – alpha diversity	Shannon = treatment age*farrow (1|sow/pig)	
16S feces – beta diversity	PC1 PC2 = age treatment*age*farrow	
ITS organ region – alpha diversity	Shannon = age organregion treatment age*organ*treat separated by organ region because of insufficient data to complete full rank analysis	
	Stomach	Shannon = age farrow age*farrow
	Duodenum	Shannon = treatment
	Jejunum	Shannon = treatment
	Cecum	Shannon = age treatment age*treatment
	Colon	Shannon = age treatment
ITS organ region – beta diversity	pc1 pc2 = age treatment farrow organsite treatment*farrow treatment*age*organsite	
	Stomach	PC1 = age
	Duodenum	PC1 = age treatment
	Jejunum	PC1 = age treatment
	Cecum	PC1 = age treatment
	Colon	PC1 = age treatment
ITS feces – alpha diversity	Shannon = age treatment sex farrow treatment*age*farrow treatment*sex*farrow (1|sow)	
ITS feces – beta diversity	pc1 pc2 = age farrow treatment sex treatment*age*farrow	

**Figure 4:**
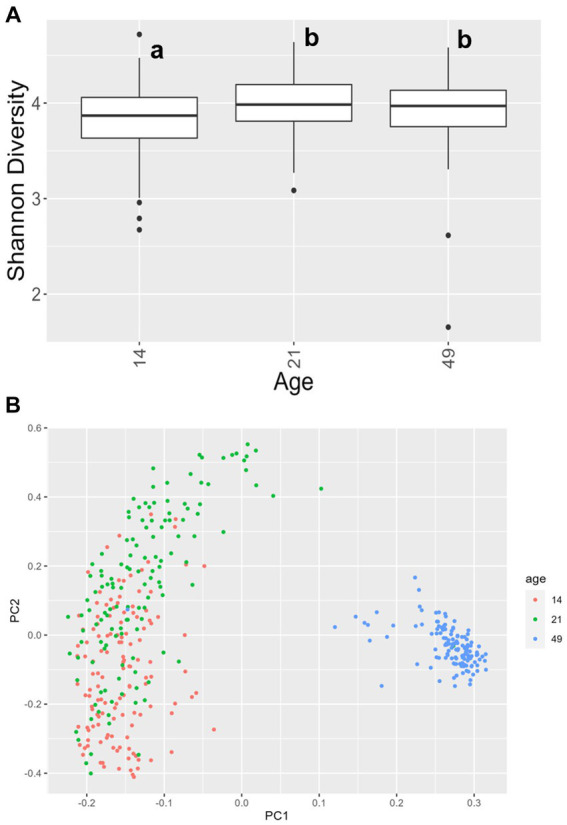
**(A)** Shannon values and **(B)** Bray–Curtis dissimilarity of the bacteriome in fecal samples by age. Day 14 was significantly lower than day 21 and day 49 for alpha diversity (*p* < 0.05), and all ages clustered independently for beta diversity (*p* < 0.05). In **(A)**, differing lowercase letters indicate significant differences between groups and dots represent outliers. (CONT: *n* = 90 piglets; PRE: *n* = 89 piglets; POST: *n* = 92 piglets; PREPOST *n* = 92 piglets).

For alpha diversity of fecal samples within the bacteriome, only age was significant, while other factors, including treatment, were not. Animals at day 14 had a lower Shannon Diversity score than animals at day 21 (*p* = 0.00030) and day 49 (*p* = 0.012), but there was no difference in alpha diversity between animals at day 21 and day 49 (*p* = 0.58; Figure 4A). For beta diversity of fecal samples, bacteriome communities were significantly different at each time point (day 14 vs. day 21, *p* = 0.0007; day 14 vs. day 49, *p* < 0.0001; day 21 vs. day 49, *p* < 0.0001; Figure 4B). In addition, significant differences were observed in farrowing group 1 vs. farrowing group 2 (*p* = 0.036).

**Figure 5:**
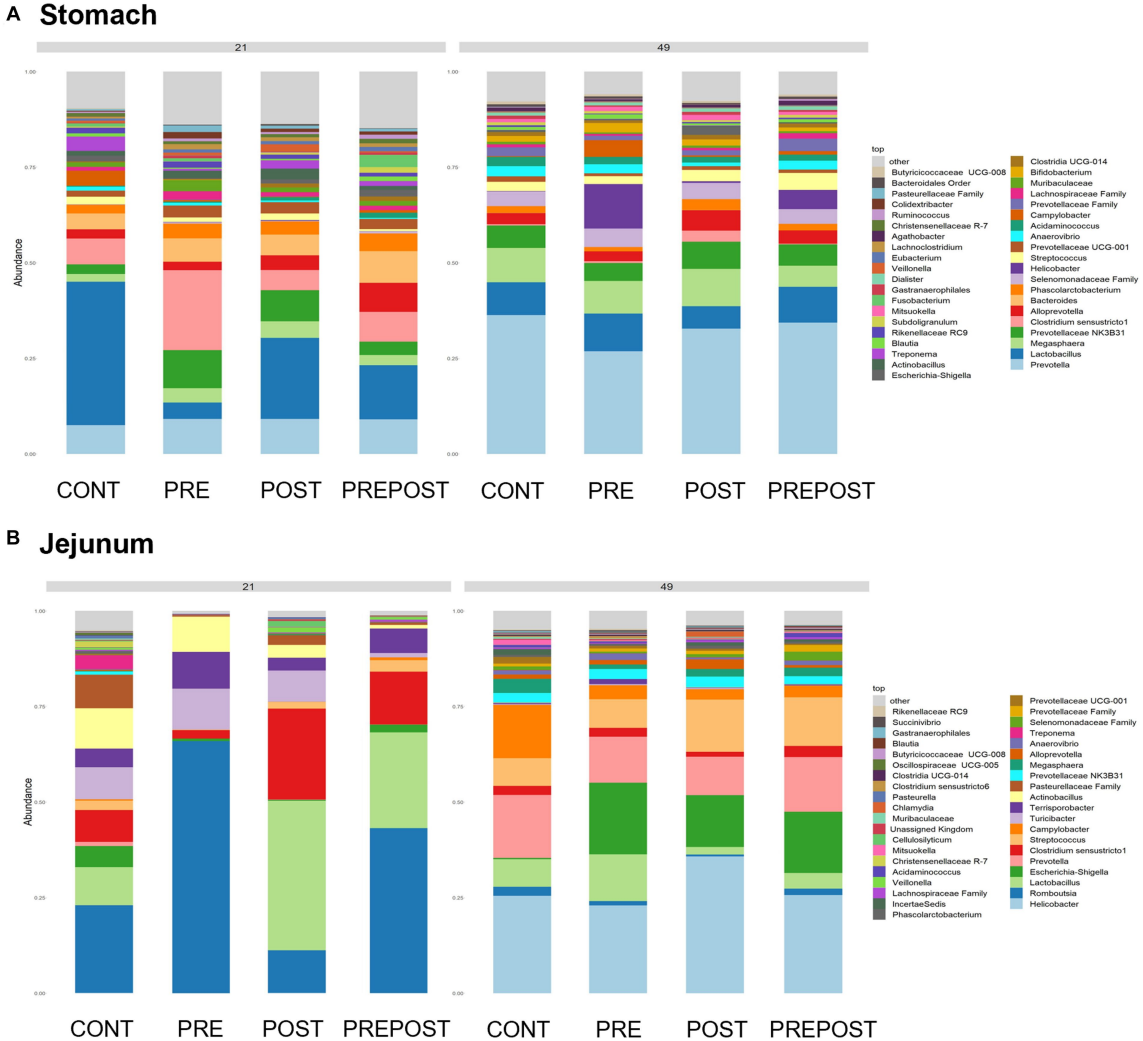
Stacked bar plot of bacterial taxon abundance of the upper GI tract including the **(A)** stomach and **(B)** jejunum. (*n* = 8 barrows per timepoint per treatment).

**Figure 6:**
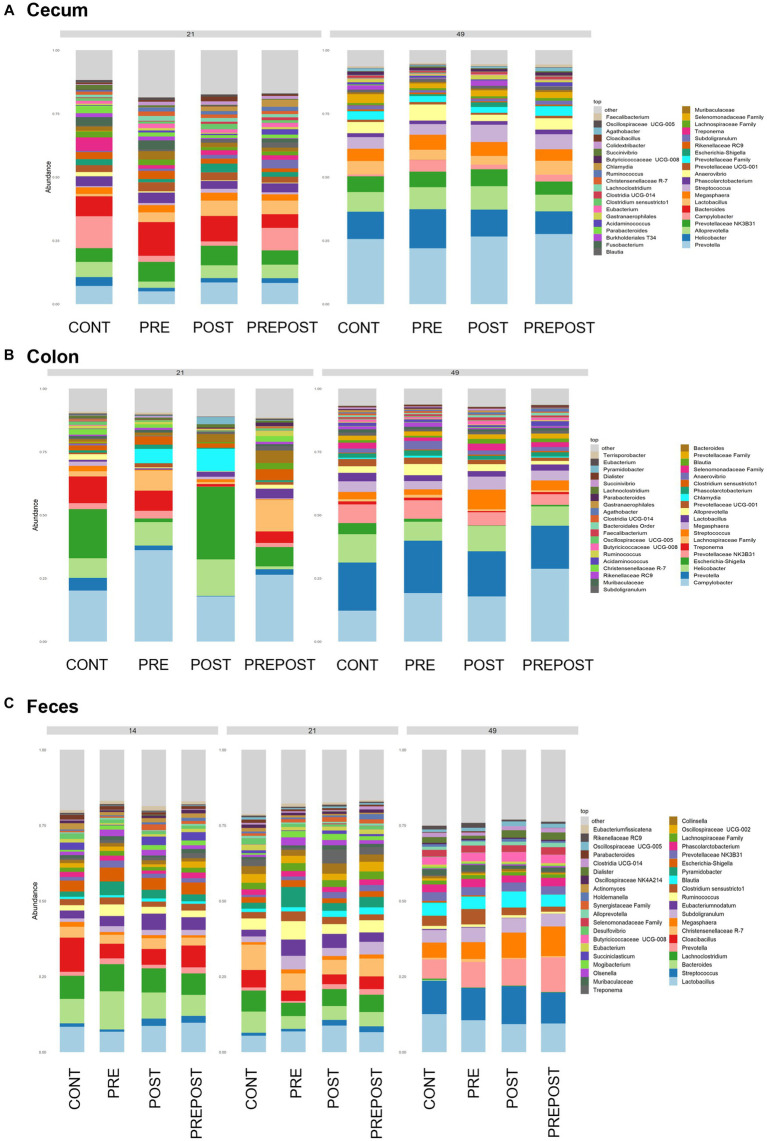
Stacked bar plot of bacterial taxon abundance of the lower GI tract including the **(A)** cecum, **(B)** colon, and **(C)** feces. (Regions of the GI tract – *n* = 8 barrows per timepoint per treatment); Feces – (CONT: *n* = 90 piglets; PRE: *n* = 89 piglets; POST: *n* = 92 piglets; PREPOST *n* = 92 piglets).

Shifts in bacteriome taxonomic composition were compared by treatment, GI organ region, and time and were analyzed by genera. It is important to note that only the 40 most abundant genera are included in the stacked barplot, while all other taxa are included in “other.” In the stomach, *Lactobacillus*, *Clostridium*, and *Bacteroides* were less prevalent in day 49 animals as compared to day 21 piglets, while *Prevotella* showed greater abundance by day 49. Interestingly, *Lactobacillus* was numerically lower in the stomach of treated animals (PRE, POST, and PREPOST) relative to CONT, but this trend was not maintained temporally (Figure 5A). In the jejunum, *Lactobacillus* was especially prevalent in the CONT, PREPOST, and POST groups, but not the PRE group at day 21, but this was again less prevalent across all groups at day 49. *Romboutsia* was also very robust in preweaned animals across all groups at day 21 only. *Prevotella*, again, was greater in weaned animals in the jejunum (Figure 5B). The cecum showed little difference in *Lactobacillus* by age or treatment (Figure 6A). Additionally, *Bacteroides* was less prevalent in day 49 animals, while *Helicobacter*, *Prevotella*, and *Streptococcus* were noticeably more abundant in day 49 animals relative to day 21 piglets. The greater abundance of *Prevotella* in day 49 animals was also seen in the colon (Figure 6B). Moreover, the colon showed a difference by treatment for *Escherichia shigella*, which was high in day 21 CONTROL and POST treatment day 21 piglets, but low in other groups, and barely detectable by day 49 in all but CONTROL animals. Figures for relative abundance for all animals can be found in Supplementary File 2.

Bacterial abundance and composition were also compared in fecal samples which were taken at day 14, day 21, and day 49 (Figure 6C). Differences by treatment were minimal. *Christensenellacaea* R-7 group was noticeably lower at day 49 relative to other timepoints. *Lactobacillus* appeared to maintain similar levels across all three timepoints. *Lachnoclostridium*, and *Bacteroides* were almost undetectable in fecal samples at day 49 despite being prominent genera at both day 14 and day 21. *Megasphaera*, *Streptococcus*, and *Prevotella* were all noticeably higher in piglets postweaning relative to both day 21 and day 14.

### Composition and diversity of the mycobiome along the GI tract and in fecal samples

**Figure 7:**
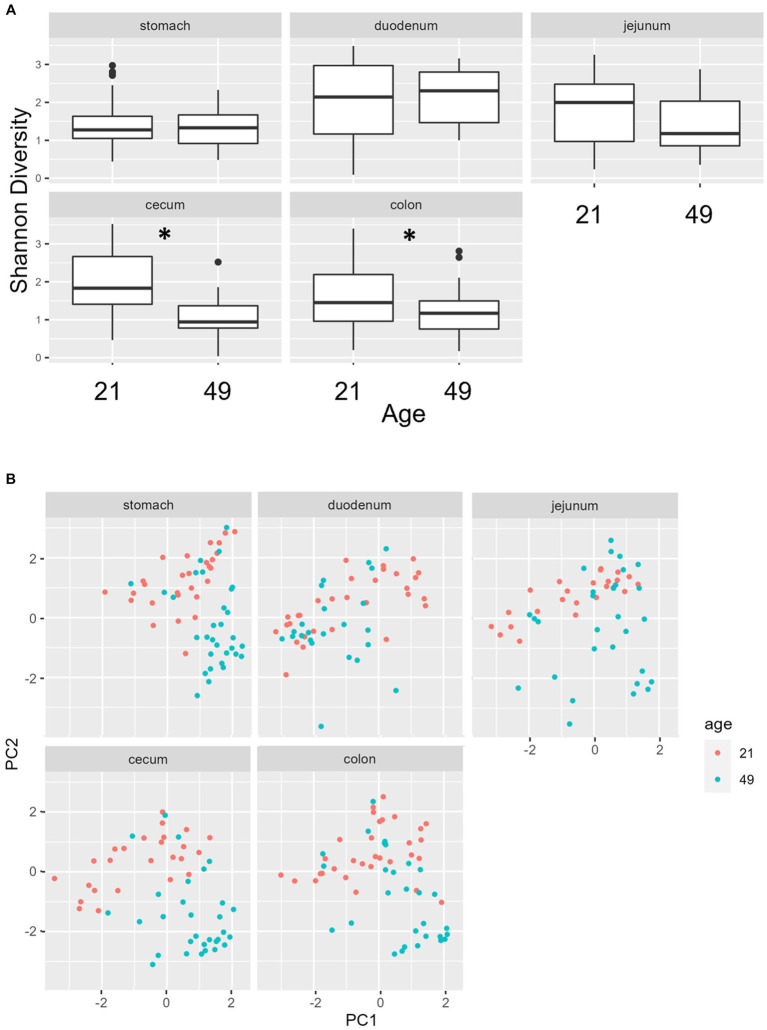
**(A)** Shannon diversity values of the mycobiome by GI organ region and age. *, significantly different between age groups within organ. Dots represent outliers. **(B)** PCA of Bray–Curtis dissimilarity of mycobiome across organs at day 21 and day 49. (*n* = 8 barrows per timepoint per treatment).

For alpha and beta diversity analyses of ITS data in regions along the GI tract, estimating greater than 2-way interactions was not feasible due to data sparsity, resulting in non-full rank design matrices. We therefore modeled these data separately within each GI organ region (please see Supplementary File 1 for all model and interaction information). For alpha diversity of ITS data, Shannon values differed statistically between day 21 and day 49 in the cecum and colon (*p* < 0.05), but not stomach, duodenum or jejunum (Figure 7A). In the cecum, the interaction between age and treatment was significant, with differences in day 21 and day 49 observed in CONT, PRE, and PREPOST treatments (*p* < 0.05), but not POST (Supplementary File 1). In the duodenum and jejunum, only treatment was significant. In the duodenum, CONT differed from POST (*p* = 0.00010) and from PREPOST (*p* = 0.00010), and POST differed from PRE (*p* = 0.015). In the jejunum, the CONT group differed from PRE (*p* = 0.0010). For beta diversity of ITS data within GI organ regions, day 21 differed from day 49 in all organ regions (stomach: *p* < 0.0001; duodenum: *p* = 0.042; jejunum: *p* = 0.0015; cecum: *p* < 0.0001; colon: *p* < 0.0001; Figure 7B). Treatment was also significant in the duodenum and jejunum. In the duodenum, the mycobiome of animals in the CONT treatment was different from that of all other groups (*p* ≤ 0.0004), as was the mycobiome in the PREPOST group (*p* ≤ 0.0026); and that in PRE and POST were comparable (*p* = 0.94). In the jejunum, CONT differed from all other groups (*p* ≤ 0.013). In the full linear model comparing main effects, Bray–Curtis values between GI organ regions were different from each other, as were farrowing groups (*p* < 0.0001).

**Figure 8:**
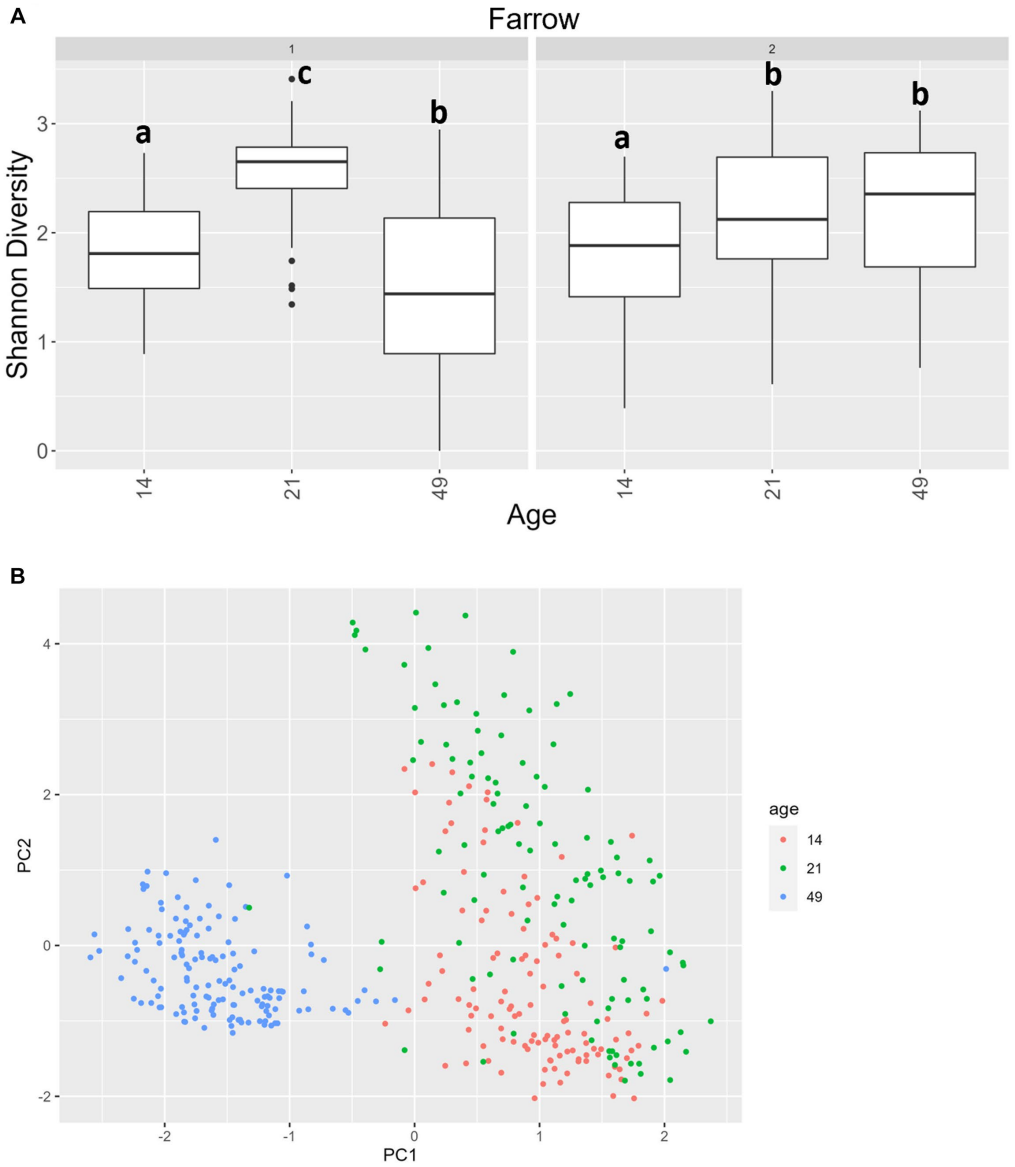
**(A)** Shannon diversity values for the mycobiome in fecal samples by farrowing group and age. All ages significantly differ from one another in farrowing groups 1 and 2, except for day 21 and day 49 in farrowing group 2, which were similar. Differing lowercase letters indicate significant differences between ages within farrowing groups, and dots represent outliers **(B)** PCA of Bray–Curtis dissimilarity of mycobiome in fecal samples. Samples were distinguishable by age (*p* < 0.05). (CONT: *n* = 90 piglets; PRE: *n* = 89 piglets; POST: *n* = 92 piglets; PREPOST *n* = 92 piglets).

For alpha diversity of ITS data among fecal samples, it should be noted that, overall, there was greater variance than there was for 16S data. Age was significant, and there was an interaction between age and farrowing group (*p* = 0.0092 and *p* = 2.2 × 10^−16^ respectively). In farrowing groups 1 and 2, all comparisons by age were different, except that day 21 was not significantly different than day 49 in farrowing group 2 (Figure 8A). For beta diversity of ITS data of fecal samples, significant terms were age (*p* < 0.0001), farrowing group (*p* = 0.0026), treatment (*p* < 0.0001), sex (*p* = 0.016), and a three-way interaction between farrowing group, age, and treatment (*p* < 0.0001). Most notably, age is the primary differentiating factor (Figure 8B). Mycobiome communities at day 21 are significantly different than communities at day 49 under all combinations of farrowing group and treatment in either PC1, PC2, or both (Supplementary File 1). The same is true for communities between day 14 and day 49. Differences between day 14 and day 21 were only present in PC2 during farrowing group 1 in the POST, PRE, and PREPOST treatments, and in farrowing group 2 within the CONT, POST, and PREPOST treatments (*p* < 0.05). Farrowing group 1 was only different than farrowing group 2 within day 21 piglets in the PRE treatment group (*p* < 0.01).

**Figure 9:**
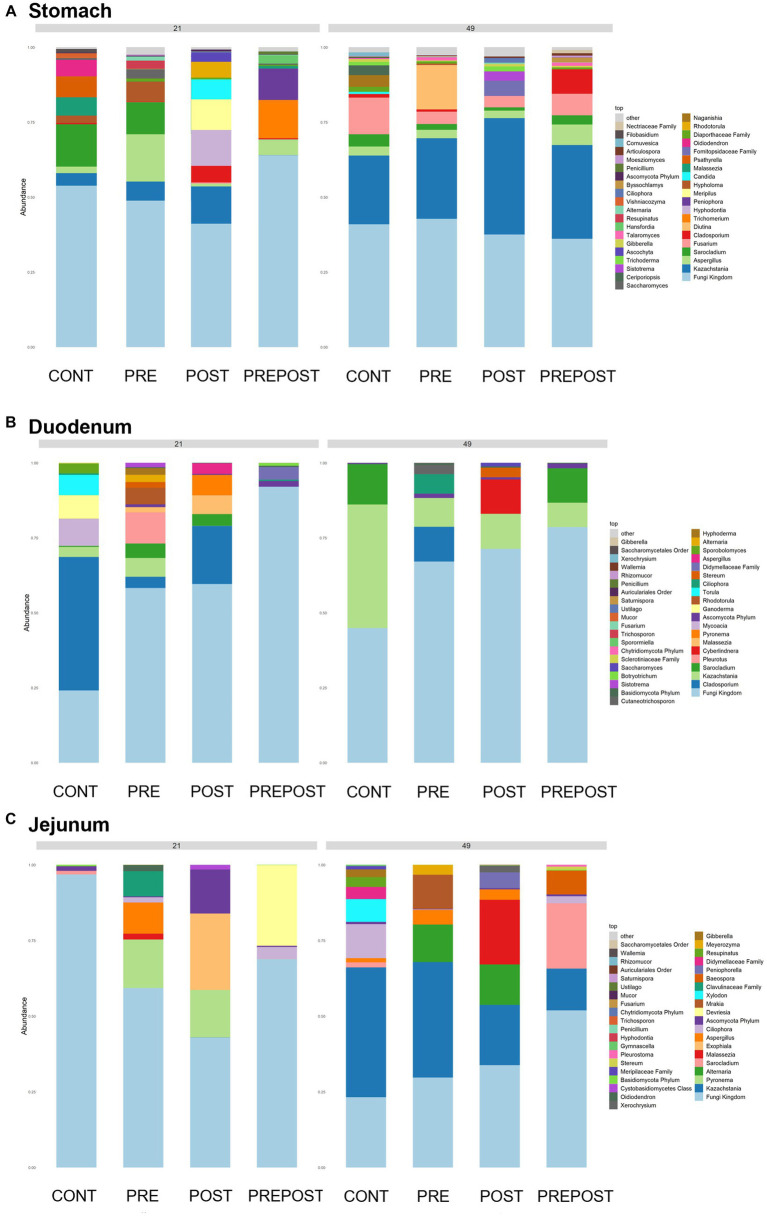
Stacked bar plot of fungal taxon abundance of upper GI tract including the **(A)** stomach, **(B)** duodenum, and **(C)** jejunum. “Fungi Kingdom” indicates unclassified fungal taxa, and “Other” indicates all taxa that are not in the topmost 40 abundant taxa. (*n* = 8 barrows per timepoint per treatment).

**Figure 10:**
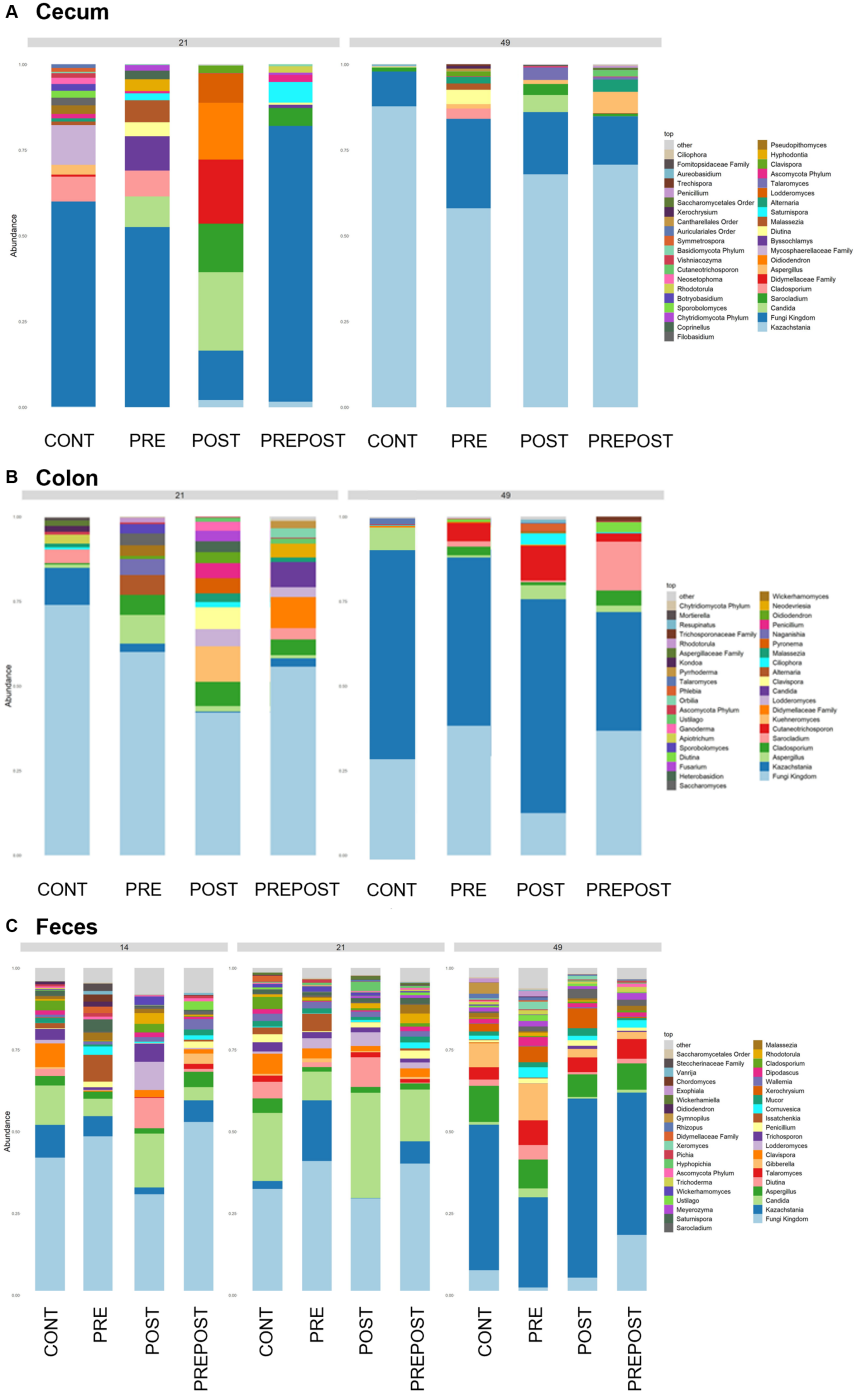
Stacked bar plot of fungal taxon abundance of lower GI tract including the **(A)** cecum, **(B)** colon, and **(C)** feces. “Fungi Kingdom” indicates unclassified fungal taxa, and “Other” indicates all taxa that are not in the topmost 40 abundant taxa. (Regions of the GI tract – *n* = 8 barrows per timepoint per treatment; Feces – CONT: *n* = 90 piglets; PRE: *n* = 89 piglets; POST: *n* = 92 piglets; PREPOST *n* = 92 piglets).

**Figure 11:**
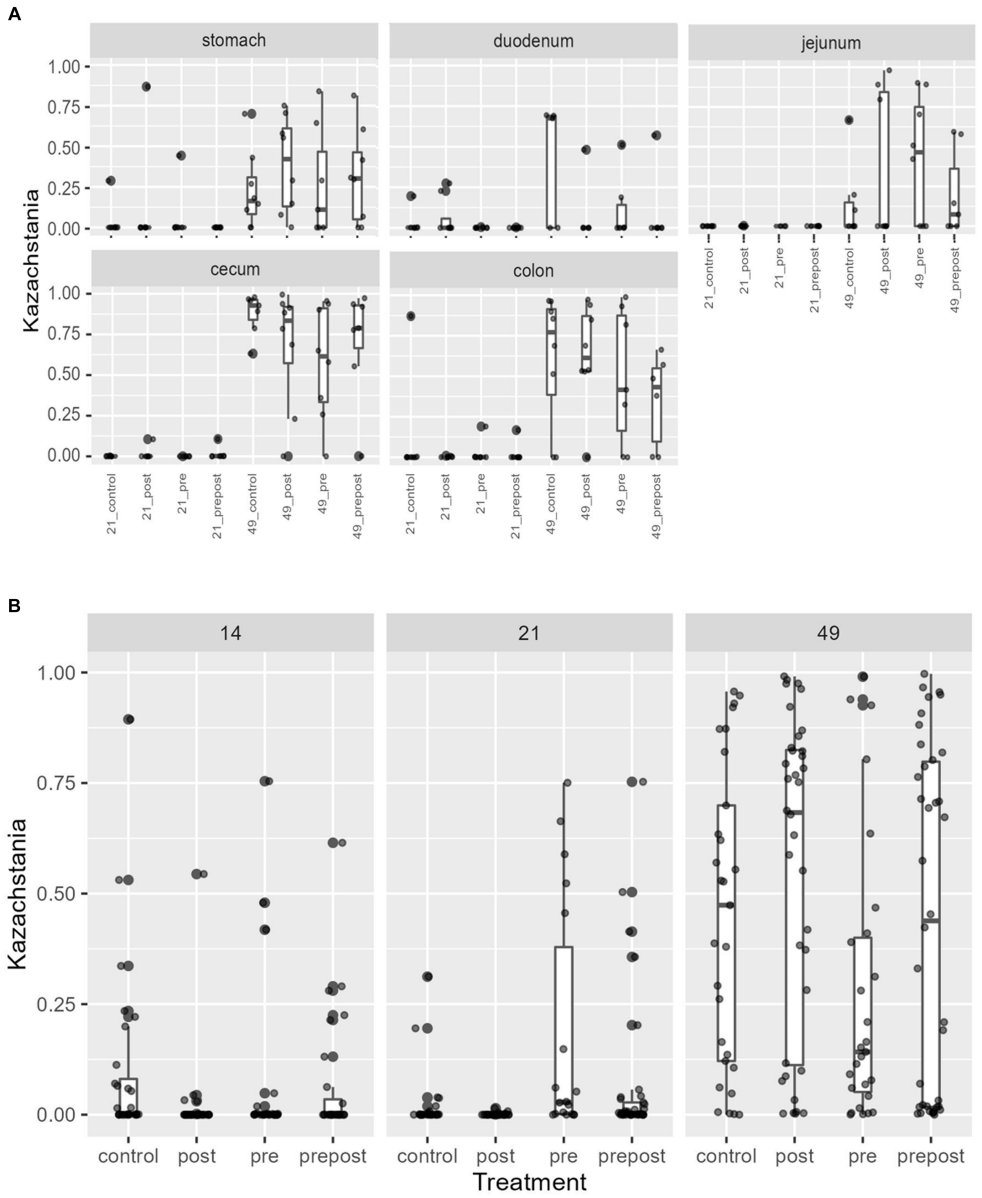
*Kazachstania* abundance in **(A)** GI organ regions and **(B)** feces by age and treatment. Dots represent outliers. (Regions of the GI tract – *n* = 8 barrows per timepoint per treatment; Feces – CONT: *n* = 90 piglets; PRE: *n* = 89 piglets; POST: *n* = 92 piglets; PREPOST *n* = 92 piglets).

In the GI organ regions, a notable fungus present in the stomach was *Aspergillus* (Figure 9A). Other genera were either lowly abundant or unclassified. In the duodenum, there were fewer fungal genera that were classifiable (Figure 9B), but day 21 piglets displayed *Cladosporium* and day 49 animals displayed *Kazachstania*. The jejunum showed high prevalence of *Pyronema* in PREPOST and POST groups at day 21, and *Devriesia* is prominent in PRE piglets. Fungal communities are broadly different by treatment at day 49 in the jejunum, but *Kazachstania* is the most abundant, with the exception of *Sarocladium* in CONT piglets (Figure 9C). Finally, the cecum and colon showed extremely high values of *Kazachstania*, but also many unclassified genera (Figures 10A,B). The same was true for feces (Figure 10C). Because *K. slooffiae* was delivered to the piglets orally for treatment, *Kazachstania* relative abundance was compared to determine if oral administration impacted relative abundance within the piglet (Figures 11A,B). Numerically, but not statistically, there was greater abundance in day 49 piglets relative to day 21 piglets for all GI organ regions which was not dependent on treatment. Moreover, there was an extremely wide variation which ranged between <5% to close to 100% in some animals.

In the feces, it is clear that the fungal population becomes more diverse and more established with age (Figure 10C). At day 14, there are mostly unclassified genera or lots of lowly abundant genera present. By day 21, the fecal population shifts to more established populations, and *Candida* and *Kazachstania* are most prevalent. The population shifts again after weaning, and by day 49, *Kazachstania* is the most abundant across all treatments. When comparing mean relative abundance of *Kazachstania* in fecal samples (Figure 11B), there was a greater abundance in day 49 piglets relative to day 21 piglets, but there were no differences between treatments. It is notable that there was a wide variation of abundance of *K. slooffiae* amongst animals like there was among regions of the GI tract.

### Antimicrobial resistance and detection of virulence genes in piglets

**Figure 12:**
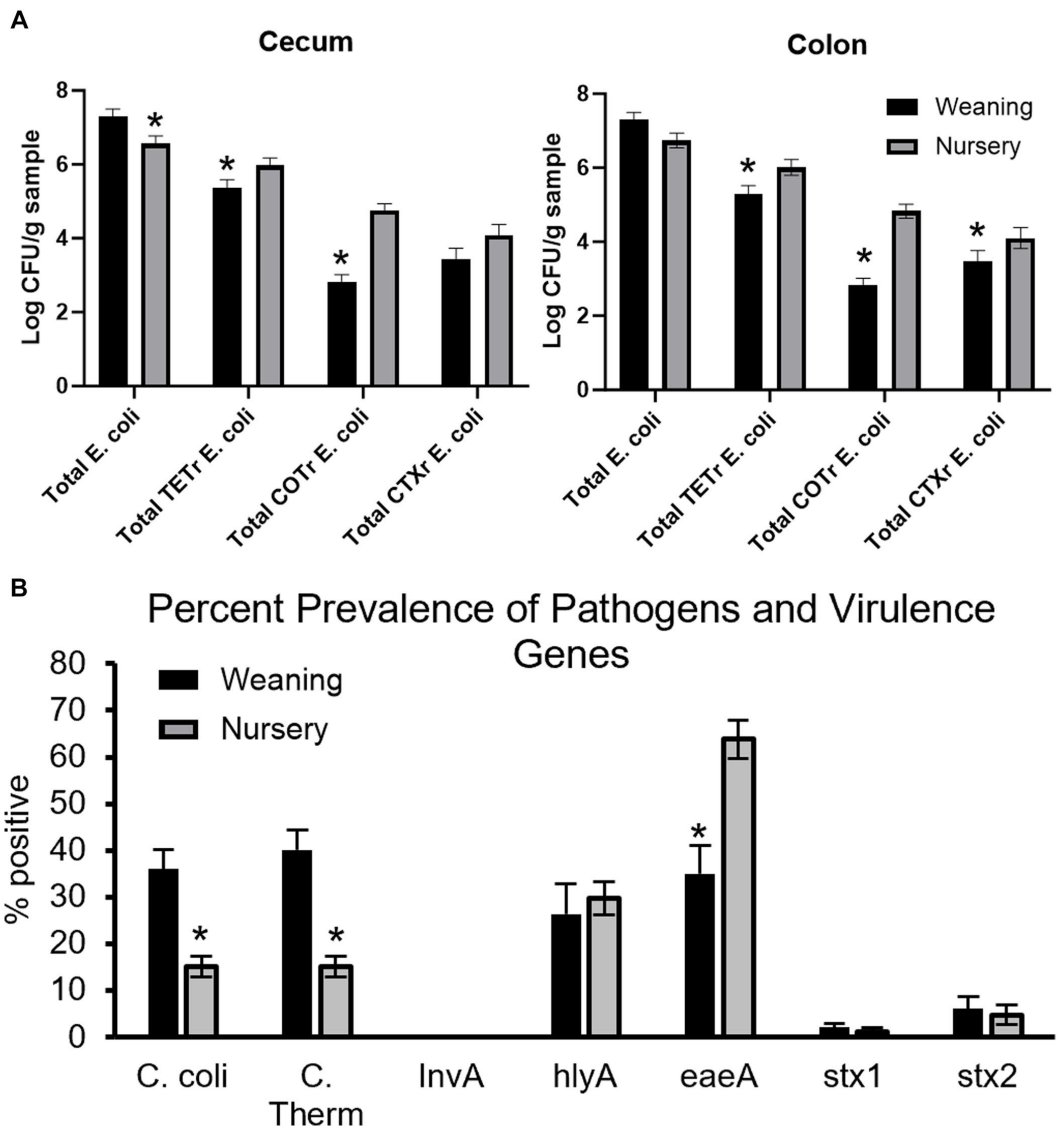
Antimicrobial resistance and detection of virulence genes in piglets. Total *E.coli* colony forming units in piglets for **(A)** cecum **(B)** and colon samples (*n* = 8 barrows per timepoint per treatment). Total CFU was calculated in piglets at weaning (day 21) and the end of nursery (day 49). **(C)** Pens were also tested for percent prevalence of pathogens and virulence genes (weaning: *n* = 32 litters; nursery: *n* = 32 litters). There were higher counts in nursery-age animals for all forms of antibiotic-resistant *E. coli* in both organs except for CTX^R^ *E. coli* in the cecum. *, significantly lower than other age group for respective microbe or gene.

Antibiotic-resistant forms of *E. coli* were enumerated from both cecal (Figure 12A) and colon (Figure 12B) contents of piglets at day 21 and at day 49. In the cecum, there were greater counts of total *E. coli* in weaning-age piglets when compared to nursery-age (*p* = 0.016), but nursery-stage pigs had greater counts of TET^R^ *E. coli* and COT^R^ *E. coli* (*p* = 0.047 and *p* < 0.0001, respectively) relative to younger piglets. There was no difference in CTX^R^ *E. coli* between age groups (*p* = 0.12). In the colon, nursery piglets had greater counts for TET^R^ *E. coli*, COT^R^ *E. coli*, and CTX^R^ *E. coli* (*p* = 0.0060, *p* < 0.0001, and *p* = 0.018, respectively). There was no difference in total *E. coli* (*p* = 0.052). No significant differences were found as an effect of treatment except for tEC_COT between control and all three treatments in the colon (*p* = 0.013). At weaning, POST piglets had greater counts than PREPOST and CONT piglets. In nursery age piglets, PRE and PREPOST piglets had greater counts than CONT piglets.

Fecal samples were also screened for detection of pathogenic microbes and genes that contribute to microbial virulence from piglets at weaning and later in the nursery (Figure 12C). Age was significant for *C. coli* (36.02 ± 4.17% to 15.06 ± 2.23% prevalence from weaning to nursery, respectively; *p* < 0.0001), thermophilic *Campylobacter* (40.10 ± 4.31% to 15.05 ± 2.23% prevalence from weaning to nursery, respectively; *p* < 0.0001), and *eaeA* (34.91 ± 6.02% to 63.81 ± 4.03% prevalence from weaning to nursery, respectively; *p* = 0.0002). Age was not significant for *InvA* (no detection at either timepoint), *hlyA* (26.37 ± 6.37% to 29.69 ± 3.54% prevalence from weaning to nursery, respectively; *p* = 0.68), *stx1* (1.99 ± 0.89% to 1.14 ± 0.89% prevalence from weaning to nursery, respectively; *p* = 0.50), and *stx2* (5.98 ± 2.69% to 4.67 ± 2.07% prevalence from weaning to nursery, respectively; *p* = 0.70). There was no effect of treatment or age by treatment interaction for *C. coli* (*p* = 0.7765 and *p* = 0.67, respectively), thermophilic *Campylobacter* (*p* = 0.89 and *p* = 0.63, respectively), *hlyA* (*p* = 0.92 and *p* = 0.058, respectively), *eaeA* (*p* = 0.66 and *p* = 0.12, respectively), *stx1* (*p* = 0.69 and *p* = 0.067, respectively), or *stx2* (*p* = 0.41 and *p* = 0.38, respectively).

### Cytokine measurements

Cytokine analysis was also conducted on serum samples of animals at day 14 and day 49 (Table 2). There was a treatment effect on IL-1β (*p* = 0.0093), IL-8 (*p* = 0.027), and TNFα (*p* = 0.022). There was an age effect on IFNγ (*p* = 0.0045), IL-1α (*p* = 0.0096), IL-1β (*p* < 0.0001), IL-2 (*p* = 0.0085), IL-4 (*p* = 0.0017), IL-12 (*p* < 0.0001), and TNFα (*p* = 0.042). There was a treatment by age interaction for IL-1α (*p* = 0.019), IL-1β (*p* = 0.0018), IL-2 (*p* = 0.040), IL-4 (*p* = 0.015), IL-8 (*p* = 0.033), and TNFα (*p* = 0.035). Significant treatment by age interactions seen were limited to differences between treatment groups at day 14 before treatments were given to animals. Only IL-1α had differences between CONT and PREPOST piglets at day 49.

**Table 2:** Cytokines assayed from serum (ng/mL).

	CONT (ng/mL)		PRE (ng/mL)		POST (ng/mL)		PREPOST (ng/mL)		*p*-values		
	Day 14	Day 49	Day 14	Day 49	Day 14	Day 49	Day 14	Day 49	Trt	Age	Trt by Age
IFNγ	3.82 ± 1.03	1.12 ± 1.36	5.69 ± 1.08	1.54 ± 2.11	3.24 ± 1.02	0.54 ± 1.31	2.19 ± 0.98	0.59 ± 1.22	0.41	0.0045	0.84
IL-1α	0.077 ± 0.018^AB^	0.022 ± 0.015^C^	0.090 ± 0.022^A^	0.029 ± 0.017^BC^	0.093 ± 0.021^A^	0.030 ± 0.016^BC^	0.0020 ± 0.022^C^	0.042 ± 0.015^ABC^	0.15	0.0096	0.019
IL-1β	0.51 ± 0.09^AB^	0.16 ± 0.088^C^	0.76 ± 0.098^A^	0.17 ± 0.093^C^	0.43 ± 0.080^B^	0.16 ± 0.077^C^	0.12 ± 0.084^C^	0.21 ± 0.072^C^	0.0093	<0.0001	0.0018
IL-1Ra	0.51 ± 0.077	0.30 ± 0.077	0.52 ± 0.082	0.36 ± 0.081	0.47 ± 0.077	0.40 ± 0.077	0.33 ± 0.073	0.36 ± 0.073	0.56	0.067	0.42
IL-2	0.43 ± 0.12^ABC^	0.12 ± 0.10^CD^	0.60 ± 0.15^A^	0.15 ± 0.12^CD^	0.55 ± 0.14^AB^	0.18 ± 0.11^CD^	0.038 ± 0.14^D^	0.24 ± 0.10^BCD^	0.21	0.0085	0.040
IL-4	1.91 ± 0.54^AB^	0.57 ± 0.44^D^	3.59 ± 0.71^A^	0.39 ± 0.49^CD^	1.83 ± 0.58^ABC^	0.67 ± 0.49^BCD^	0.051 ± 0.76^CD^	0.86 ± 0.44^BCD^	0.11	0.0017	0.0154
IL-6	0.14 ± 0.038	0.041 ± 0.042	0.16 ± 0.042	0.067 ± 0.048	0.17 ± 0.043	0.046 ± 0.042	0.016 ± 0.043	0.091 ± 0.040	0.51	0.056	0.083
IL-8	0.022 ± 0.010^B^	0.031 ± 0.011^B^	0.082 ± 0.013^A^	0.031 ± 0.011^B^	0.034 ± 0.011^B^	0.038 ± 0.011^B^	0.023 ± 0.011^B^	0.027 ± 0.010^B^	0.027	0.26	0.033
IL-10	0.63 ± 0.17	0.30 ± 0.18	0.67 ± 0.17	0.29 ± 0.17	0.63 ± 0.18	0.26 ± 0.18	0.086 ± 0.17	0.38 ± 0.17	0.46	0.13	0.16
IL-12	0.65 ± 0.057	1.054 ± 0.057	0.73 ± 0.060	1.14 ± 0.060	0.71 ± 0.057	1.0024 ± 0.057	0.65 ± 0.55	1.10 ± 0.055	0.49	<0.0001	0.58
IL-18	1.14 ± 0.22	0.70 ± 0.22	1.021 ± 0.24	0.71 ± 0.24	1.10 ± 0.22	0.63 ± 0.22	0.40 ± 0.21	0.76 ± 0.21	0.40	0.18	0.19
TNFα	0.059 ± 0.036^B^	0.046 ± 0.034^B^	0.23 ± 0.037^A^	0.049 ± 0.037^B^	0.061 ± 0.035^B^	0.040 ± 0.037^B^	0.034 ± 0.033^B^	0.044 ± 0.032^B^	0.022	0.042	0.035

Values are reported as least squares means ± standard error (SEM). Groups not connected by same letter are significantly different (*p* < 0.05). Although there were some significant interactions between treatment and age for some cytokines, most all of these were only significant at day 14, but not at day 49, when an effect of treatment should have been detected.

## Discussion

In this study, feeding *K. slooffiae* to piglets before and during the weaning transition was studied to determine impacts on health and growth performance. *K. slooffiae* has been consistently observed as a commensal in the pig gastrointestinal tract and has shown positive interactions with beneficial commensal bacteria *in vitro* (13–16, 22, 42); thus, we hypothesized that feeding *K. slooffiae* as a probiotic would improve piglet performance through: (a) indirect modulation of the combined microbiome (13); or (b) direct effects due to increased abundance of *K. slooffiae* in the gastrointestinal tract (18, 19). Overall, we did not observe any improvements in piglet health or growth as an effect of *K. slooffiae* treatment, nor any detectable, sustained differences on alpha and beta diversity within either the bacteriome or mycobiome at day 49 as a direct result of supplementation. However, we documented spatial dynamics within the piglet GI tract alongside temporal changes in the fecal mycobiome through the weaning transition for the first time. This revealed that diet and region of the GI tract of the piglet are the most important factors which influence the mycobiomes of the GI tract and feces as determined by alpha and beta diversity metrics.

Several prior studies examined *K. slooffiae* as a potential treatment to alleviate the impacts of postweaning stress. Similar to this study, there was little to no improvement in growth performance or global health among piglets, regardless of experimental differences such as a later weaning date (D27-28 compared to day 21 here) and multiple, consecutive days of administration of *K. slooffiae* postweaning. In concordance with results from this study, age was the most important contributor to differences in alpha and beta diversity, followed by litter. However, a temporary increase in alpha diversity of the bacteriome and increased short chain fatty acid production as detected from fecal samples following treatment was observed (18). Another report of feeding *K. slooffiae* to piglets was in the context of confirming a metabolic mechanism previously established in a neonatal piglet jejunal cell line (42). There, they found *K. slooffiae* contributed to lysine desuccinylation-mediated glycolysis, which was confirmed *in vivo* using intestinal tissue collected from three-week old piglets administered a daily oral gavage of *K. slooffiae* for 3 weeks. Further, this group found an increase in villus height as well as ratio of villus height to crypt depth in the jejunum and ileum of these animals, but they did not compare microbiomes of the treated piglets. Taken together, these studies suggest that *K. slooffiae* may contribute to host health by improving metabolism locally within the GI tract but may not be transferable to other global parameters, such as growth. Further studies which correlate microbiome communities with metabolic parameters within the gut following *K. slooffiae* administration are needed.

In the current study, it is worth noting that dosage and administration frequency of *K. slooffiae*, as well as collection timepoints following administration, were different than the aforementioned studies. Here, frequency of *K. slooffiae* administration was selected, in part, due to feasibility of implementing this method into production models. As piglets are already stressed during the weaning transition and administration takes time and handling, orally gavaging piglets daily is not practical. Additionally, the previous studies (18, 42) both reported higher CFU in their dosages and more frequent administration than those used here. Here, if sample collection and treatment overlapped on the same day, samples were collected from pigs before treatment. Therefore, any samples taken were at least 7 days after administration depending on the sampling day (e.g., POST piglets sampled on day 21 received their supplementation dose 7 days after their previous sampling, while POST piglets sampled on day 49 were 4 weeks past administration). Thus, any metabolic or microbial changes due to administration may have been transient and not detectable by the sampling date. Therefore, sampling at a closer interval after administration of *K. slooffiae* may have revealed transient bacteriome and/or mycobiome changes. It is difficult to permanently manipulate the microbiome through the use of treatments, feeding or otherwise, as such changes in microbiome diversity and short-chain fatty acid production appear to be short-lived, and the initial microbial community may be reestablished when no additional pressures are exerted (18, 43). One contradictory report concluded that 3 weeks of carbadox, an in-feed antibiotic in the swine industry, administered during the weaning transition had a long-term influence on the bacteriome (44), but no alternatives have been identified with a similar impact. Given that the microbiome in weanling piglets is in flux because of age and change in diet, it may be more sensitive to disturbances that are inherent to growth and current swine production systems when compared to an older animal, where significant perturbations such as an intensive course of antibiotic treatment is required to see lasting results (45).

We have previously documented the temporal characterization of colonization of *K. slooffiae* using fecal mycobiome analysis of piglets through the weaning transition (16), as well as GI organ regions and feces concurrently at a single timepoint (day 35) (13), but this study was able to expand those to include the characterization of *K. slooffiae* in both multiple GI organ regions and feces at multiple timepoints. This taxon is the dominant species of *Kazachstania* in the piglet around the weaning transition (16). *K. slooffiae* has been detected as early as 3 days after birth (46), and abundance peaks shortly after weaning (14), but decreases as the piglet matures past the growing phase into adulthood (15). Previously, we collected samples at day 35 (weaned at day 21), and *K. slooffiae* was indeed a dominant fungal community in the mycobiome at this time (14). Here, we found *K. slooffiae* well established in GI organ regions and fecal samples collected from piglets at day 49, but lowly abundant in most fecal samples and almost undetectable in organ regions at day 21 when the piglets were weaned. Based on our past work, we have been able to detect *K. slooffiae* as early as 1 week after the transition to a grain-based diet after weaning (16). Others have been unable to detect *K. slooffiae* before the nursery period (15), thus indicating the possibility that *K. slooffiae* abundance increases in response to dietary changes after weaning.

Mycobiome changes are largely a factor of diet and the environment. Fungal species change drastically up to and through the weaning transition and resemble the mycobiome of adult pigs as early as day 24–35, and mycobiome clustering by GI organ region is not apparent at day 35 (13). Additionally, most fungal taxa are likely transient and never fully colonize the gut; a review of human mycobiome research found many species only appeared in a single study or even a single sample among time-course trials (47). Humans and pigs share many physiological similarities, including digestion (48), and fungi may be transient in a similar nature here. This short inoculation may be due, in part, to fungi present in the feed, as many taxa are foodborne and can transiently colonize the gut (49, 50), but we have previously been unable to detect fungi from colostrum, mature milk, sow feed, or creep feed through either culturing or amplification (16). Because piglets experience a dietary change during weaning from milk to solid feed, and all piglets in this study were subjected to the same transition at the same time, it is highly likely that changes in the mycobiome between day 21 and day 49 were due to this shift. There are few studies assessing mycobiome changes in piglets; however, several studies indicate that the bacteriome is more highly impacted by changes in diet than by age. Piglets that are weaned onto solid food before littermates will experience changes in the bacteriome separate from their littermates (51), and the bacteriome shifts at the time of weaning whether it is 14, 21, or 28 days (52). Fungal communities likely experience similar transitions in response to diet or because of bacteriome changes. As the piglet makes the transition from a milk-based diet to a plant-based solid diet, certain fungal communities can grow in response to environmental and dietary changes, either directly through the addition of a more favorable energy source or indirectly through the shift in bacteriome, especially when considering dietary carbohydrates (53). A study on human diets concluded that *Candida* was positively correlated with carbohydrate consumption (54), and starch degradation (55), both of which are needed by postweaning piglets that have transitioned to a plant-based diet from a milk-based diet. *K. slooffiae* is closely related to *Candida* (56) and likely shares similar mechanisms for carbohydrate metabolism. Beyond nutrition, fungal growth and subsequent inhabitation in the piglet are also likely connected to the microenvironments produced within individual farrowing crates. Most swine systems house dams and their litters in crates for the duration of lactation, separate from other litters, and changes that take place here may contribute to the mycobiome of the piglets. For example, differences in temperature, humidity, and air velocity can vary drastically between crates within the same farrowing room at any given time (57), which may influence fungal populations and subsequent GI tract colonization.

There was a great deal of individual variation in the abundance of the genera *Kazachstania* in these piglets by GI organ region as well as fecal samples, which ranged from less than 5% mean relative abundance in some animals to close to 100 percent in others. A prior study showed piglets from some sows did tend towards similar abundances of *K. slooffiae* (46). However, in other sows, there was still a great deal of variation in relative abundance of *Kazachstania* amongst piglets within the same litter. We also previously found a high variation in the relative abundance of the family *Saccharomycetaceae* (*K. slooffiae*) despite all piglets having the same feed and housing after weaning (14), and others have reported similar findings (46). This may be due to differences in colostrum consumption or variation in milk composition between teats in the sow. While diet and environment may have been comparable after weaning, milk composition and quantity varies markedly between sow at all stages of lactation (58), even by teat placement (59, 60). Importantly, piglets must rely on passive immunity until their own immune system is developed, which can take weeks after birth (61), and colostrum consumption is associated with early establishment of the immune system that persists as seen by immunological indicators at weaning (62, 63). Piglets that do not receive an adequate volume of colostrum, which is generally accepted as 200–250 g after birth (64), can suffer from reduced immune response among other repercussions (63). Colostrum consumption in piglets both within and between litters is rarely uniform because many factors can impact it, such as birth weight and litter size (65, 66). Colostrum can shape the microbiome by preferentially selecting for microbiome constituents through both nutrition and immunological responses of the host. Therefore, variation in colostrum consumption can impact piglet immunity and gut microbiome establishment and may explain the wide variation in abundance of certain microbes such as *K. slooffiae* in the GI tract. Moreover, piglets are coprophagic and can consume, on average, 20 g of fecal material a day, including from the sow (67). *K. slooffiae* is not commonly found in environments outside the digestive systems of large mammals (56, 68, 69). Therefore, the sow is the most likely source of transmission to piglets and is a strong indicator of the piglet mycobiome (46). Piglets engaging in different rates of coprophagy may have contributed to the differences seen here in addition to biological variability and differences in colostrum consumption.

Another possible contributor to variability seen here was sample size. Our past bacteriome and mycobiome studies [13 and 14] had small sample sizes and took place in the same farrowing group. As the current study was scaled up to incorporate more animals, the experimental design was more complex and therefore may explain differences. Indeed, sow accounted for a moderate to high proportion of random variance in all beta-diversity analyses where sow was included (i.e., GI organ region analyses did not consider random variance due to sow because only one barrow per sow was sampled per timepoint). Future studies will aim to increase sample size in order to reduce variability attributed to sow, ideally in the same farrowing room and during the same farrowing instance. Sow mycobiomes were not sampled and sows could not be repeated across farrowings; therefore, we cannot determine whether variability in results was due to sow effect or farrowing specifically. Environment within crate may have contributed because sanitation methods used can impact piglet bacteriome (70). However, as mentioned previously, microbes from the sow greatly influence the piglets (46) and likely had a greater impact. This variability in diversity also likely contributed to significant differences observed among treatment groups of regional GI organ mycobiome samples. For example, significant differences in mycobiome alpha diversity were observed between CONT and POST groups at day 21 in the cecum. However, the POST treatment was not administered until day 21 after samples were taken; therefore, no significant differences should have been observed and can likely be attributed to another source other than treatment. This was the first study to investigate the mycobiome by region of the GI tract over time and treatment with a fungal probiotic candidate; therefore adequate sample sizes were unknown, but future experiments will be designed with a greater number of piglets from an increased number of sows.

Past reports of spatial GI tract analysis in piglets revealed very distinct separation between upper and lower GI tract bacteriomes. Liu and colleagues found that spatial bacteriome analysis clustered the duodenum and jejunum together, the ileum by itself, and the cecum, colon, and rectum as a unit when comparing bacteriome beta diversity. They observed more volatility in the small intestine over time (i.e., Shannon diversity indices decreased until midway through lactation and then increased prior to weaning followed by another decrease into maturity) when compared to the large intestine (remained relatively constant throughout life) (71). In the present study, bacteriome alpha diversity was not as distinct between GI organ regions; it was lowest in the colon and jejunum compared to the stomach and cecum at day 21, but by day 49, diversity within the colon increased to match that of the stomach and cecum (Figure 2B). Bray–Curtis dissimilarity was a better distinguisher because bacteriome communities in each region of the GI tract were distinct from all others at day 21 except for the jejunum and stomach which could not be differentiated (Figure 2B and Supplementary Table S1). At day 49, communities in the jejunum, cecum, and colon were distinct from each other, but the stomach shared similarities with both the cecum and colon. The high Shannon diversity in the stomach was an interesting finding, as past reports have found low Shannon diversity in the stomach bacteriome and increasing Shannon diversity as it progresses along the GI tract, with colon and feces being the highest (13). Other studies in the piglet also found increasing Shannon diversity along the GI tract in postweaned mucosa – different than the GI organ regions sampled in the current study – and low Shannon diversity in the mucosa of the stomach (72). One study found that Shannon diversity was higher in the collective small intestine relative to the collective large intestine, especially throughout the weaning transition (71). These authors also note extreme instability in the small intestine relative to the large intestine. It is unclear why the stomach would be similar to the caudal GI tract here, but dynamic changes due to early life and weaning combined with differences in sampling technique between our study and previous studies may have contributed, thus making it difficult to generate a definitive conclusion regarding Shannon diversity in the young pig.

We have previously reported increasing Shannon alpha diversity values with less variability over time in the fecal bacteriome of piglets through the weaning transition (15, 16). The current study found that alpha diversity of the fecal bacteriome increased between day 14 to day 21 and remained constant between day 21 and day 49. However, beta diversity between all three age points were different, indicating that although Shannon diversity remained constant between day 21 and day 49, taxonomic changes occurred and are the primary driver of diversity changes. Previously, others found a plateauing of alpha diversity after weaning (73). While the aforementioned study revealed a small increase in alpha diversity in samples taken between day 21 and day 42, alpha diversity of samples taken at day 42 were similar to that of day 21 samples. The same was not true for beta diversity, as taxonomic variation was apparent in bacterial abundance stacked bar plots, and they found diet to be the primary driver of bacteriome changes between birth and day 42. This change in composition is not surprising. Given the differences in milk composition over the course of lactation, the early consumption of sow feed prior to weaning, and subsequent transition to nursery feed, the flexibility of the gut bacteriome is expected, and the diversity is likely an advantage as microbes are selective for particular substrates. Alpha diversity of the fecal mycobiome does not appear to follow a linear trend across the weaning transition, likely due to the strong environmental impact. While we have previously reported a decrease in Shannon values over time in piglet fecal samples (14), that study was limited to three litters, and other data shows the mycobiome is extremely volatile during this time period (16). A past study in children also saw no temporal pattern in alpha diversity of fungal populations over a more extended dietary and developmental shift (i.e., alpha diversity decreased from birth to 6 months and increased again between six- and eighteen-months) (74). Viewing taxonomy over time was far more informative, as this also revealed taxonomic composition shifts even if alpha diversity was not different between groups.

As others have recorded in the past, *Lactobacillus* was less prevalent in the stomach of older animals at day 49 when compared to newly weaned piglets at day 21. Interestingly, the abundance of this microbe in feces was relatively similar across all three timepoints. *Lactobacillus* is closely related to milk digestion, and as the animal makes a transition to a grain-based, carbohydrate-heavy diet, the needs of the host change to reflect that. After piglets are weaned and are no longer receiving nutrition in the form of milk, the abundance of *Lactobacillus* decreases, and other bacteria such as *Clostridium*, *Prevotella*, *Proteobacteriaceae*, and *E. coli* can take its place (73, 75). Indeed, older piglets in this study had greater abundance of *Prevotella* in all regions of the GI tract and in fecal samples, which likely allowed them to better digest fermentable dietary fiber and complex dietary polysaccharides (76). *Bacteroides* were also less prevalent in older animals; *Bacteroides* is common in bacteriomes of those with diets that consist of high protein and high animal fats, both of which are highly prevalent in milk (77). Many microbes are responsible for the production of volatile and short-chain fatty acids needed to accommodate the metabolic needs of the epithelium, and bacteria that do this such as *Phascolarctobacterium* were less prevalent in older animals. The decrease over time with a simultaneous increase in other species like *Prevotella*, another microbe responsible for production of short-chain fatty acids, suggests a shift to fill this niche role while also accommodating the shift in available nutrients. Additionally, the genus *Romboutsia* appeared to be isolated to the jejunum of day 21 piglets. While a relatively recent discovery, this genus is a highly dynamic group with the ability to metabolize carbohydrates, ferment amino acids, and synthesize vitamins depending on the species when analyzing isolates from humans (78); they may be especially useful during the transition period to accommodate changes in diet.

In addition to the microbiome analysis completed, GI organ region and fecal samples were also tested for the presence of antibiotic-resistant *E. coli* and genes associated with virulence, respectively. As the intention of *K. slooffiae* administration was to serve as an antibiotic alternative, these analyses were conducted to determine the influence on metrics associated with antibiotic resistance. Similar to the microbiome analysis, any treatment effects were likely due to age and environment (e.g., weaning-age piglets in the POST treatment group had greater levels of COT^R^ *E. coli* than weaning-age piglets in the CONT group, and this was before any treatment had been administered). All forms of antibiotic-resistant *E. coli* were detected at higher rates in nursery animals when compared to samples taken from younger animals. Recently, another group found that tetracycline resistant genes increased until weaning in piglets and remained stable into adulthood (52), which is consistent with what was found here. Potential pathogens in fecal samples were detected using molecular approaches. Several virulence genes are associated with *E. coli* pathotypes, and amongst the genes assayed, *eaeA* (*E. coli* attaching and effacing gene) was significantly more prevalent in nursery-age animals, while other *E. coli* genes that specifically encode for Shiga toxin were found to be low at all ages. Other *E. coli* virulence genes exhibited had no differences between groups (*hlyA*). Diarrhea scores were not recorded for these animals as no animal was symptomatic enough to require a medical report or veterinary intervention, but higher expression of *eaeA*, especially when combined with antibiotic-resistant forms of *E. coli*, can increase intestinal diarrhea by producing lesions on the epithelial cell membrane (79). Additionally, *C. coli*, a commensal in swine, was lower in nursery animals relative to weaned animals. Past literature has reported prevalence as high as 100% in swine herds, and it can contribute to enteritis as well (80). *Salmonella* as determined by targeting the *invA* gene was not present at detectable levels in these piglets. Antibiotic resistance on swine farms has been well-documented, due in part to the use of antibiotics as growth-promoters, and recent initiatives to control antibiotic use to slow antibiotic resistance have been put in place globally (81). As these piglets were not exposed to antibiotics due to the nature of this study and were kept in separate pens from other piglets that may have been treated, this data yielded information about this unit’s herd as a whole and the prevalence of antibiotic resistance at this location.

Finally, a number of cytokines were compared for animals at day 14 (before any treatments were administered) and day 49 (4 weeks after the last treatment given) from serum samples to determine if administration of *K. slooffiae* could have an effect aside from improving the metabolism of cells within the intestinal lining (42), such as improved immune function from a systemic level. For almost all cytokines where there was a treatment by age interaction, there were no differences between treatments at day 49, indicating *K. slooffiae* administration likely does not have any long-term effects on systemic immunological response in piglets. There were some differences between treatment groups at day 14. Since this sampling took place prior to administration of PBS or *K. slooffiae*, we are attributing these differences to sow effect, similar to some of the differences seen in the microbiome before treatment. The intestinal immune environment is not fully developed in the piglet until after most commercial farms wean between 21 and 28 days of age (82), so any impacts on systemic cytokine prevalence, possibly those associated with the innate immune response, may have occurred in the short-term and not been apparent by day 49 when the later blood sample was taken.

## Conclusion

No remarkable changes were found in any growth parameters, health parameters, or microbiome diversity metrics following the administration of probiotic candidate *K. slooffiae*. Furthermore, abundance of *K. slooffiae* within the GI tract of treated animals was not impacted. Instead, we revealed that changes due to the weaning transition (i.e., diet and environment) on the piglet bacteriome and mycobiome were instrumental in shaping microbial communities before, during, and after this critical timepoint. The mycobiome has been documented along the GI tract previously and at discrete timepoints, but this research characterized the piglet GI tract mycobiome through the weaning transition for the first time in a large-scale piglet study, thus providing a foundation for future mycobiome-oriented investigations. Farrowing group affected some analyses, but this is likely due to either sow or environmental factors, and we recommend including more sows within the same farrowing group to reduce experimental variability in the future. Based on these findings, *K. slooffiae* delivered at this dosage and concentration may not be an effective alternative growth promotant or method to support piglet health.

## Data availability statement

The data presented in the study are deposited in the NCBI repository, accession number PRJNA1020867.

## Ethics statement

The animal study was approved by USDA-ARS Institutional Animal Care and Use Committee of the Meat Animal Research Center. The study was conducted in accordance with the local legislation and institutional requirements.

## Author contributions

KH: Formal analysis, Writing – original draft, Writing – review & editing, Visualization. KS: Conceptualization, Data curation, Formal analysis, Funding acquisition, Investigation, Methodology, Project administration, Resources, Supervision, Writing – original draft, Writing – review & editing. WO: Conceptualization, Data curation, Formal analysis, Funding acquisition, Investigation, Methodology, Resources, Supervision, Writing – review & editing. JW: Conceptualization, Data curation, Formal analysis, Funding acquisition, Investigation, Methodology, Resources, Supervision, Writing – review & editing. MC: Methodology, Writing – review & editing. BN: Methodology, Writing – review & editing. LR: Methodology, Writing – review & editing. IR: Data curation, Formal analysis, Writing – review & editing. TR: Writing – review & editing. CD: Writing – original draft, Writing – review & editing, Formal analysis, Methodology, Supervision, Visualization.
