# Supplementary material for: Weaning transition, but not the administration of probiotic candidate Kazachstania slooffiae, shaped the gastrointestinal bacterial and fungal communities in nursery piglets
Source: Front Vet Sci. 2024 Jan 11;10:1303984. doi: 10.3389/fvets.2023.1303984 (PMC10808496; doi:10.3389/fvets.2023.1303984)

Figure S1. Shannon diversity of mycobiome organ samples showing alpha diversity by organ region, treatment, and age.

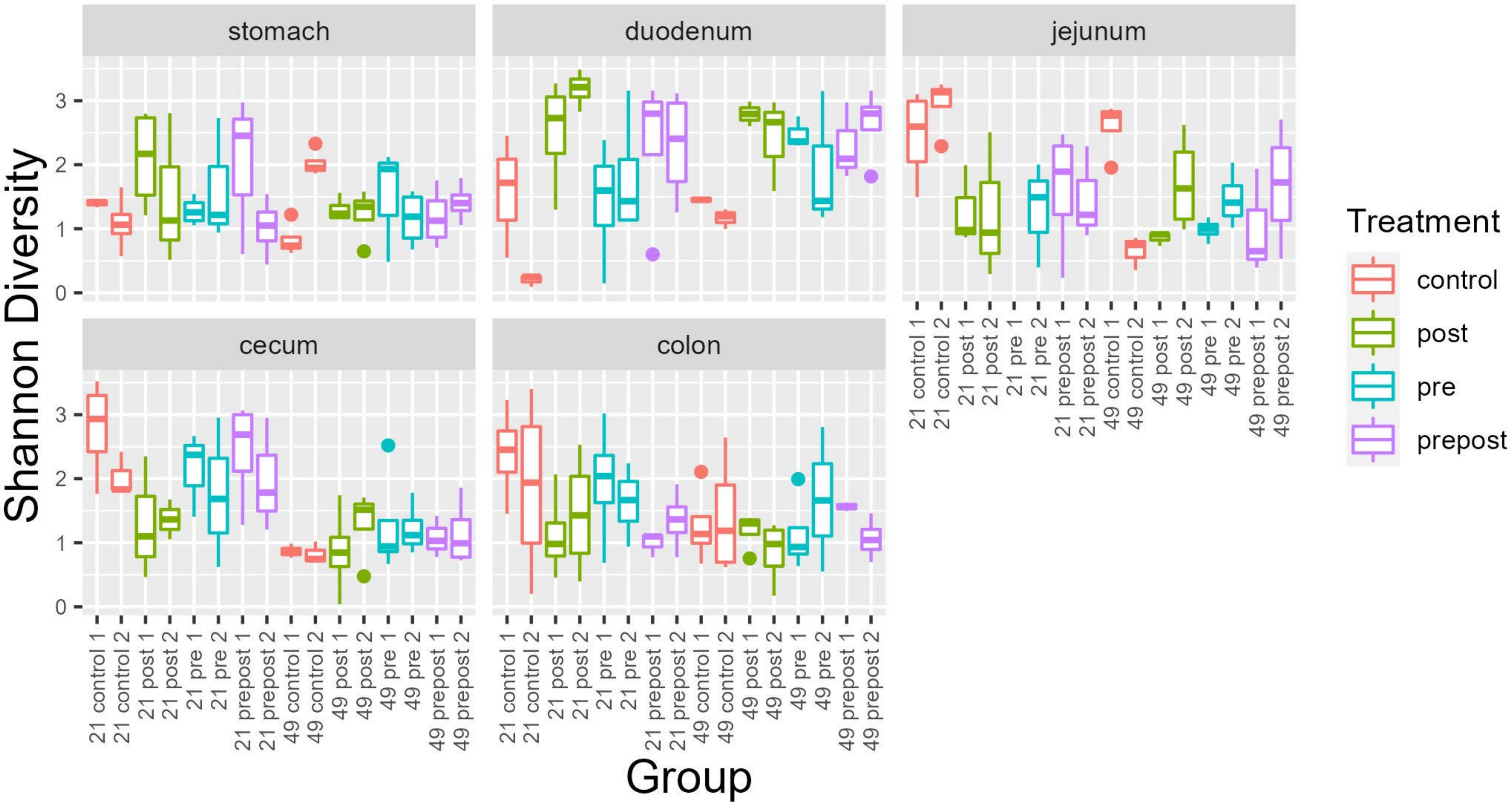

Figure S2. Stacked bar plot of bacterial taxon abundance within stomach samples for all piglets sampled.

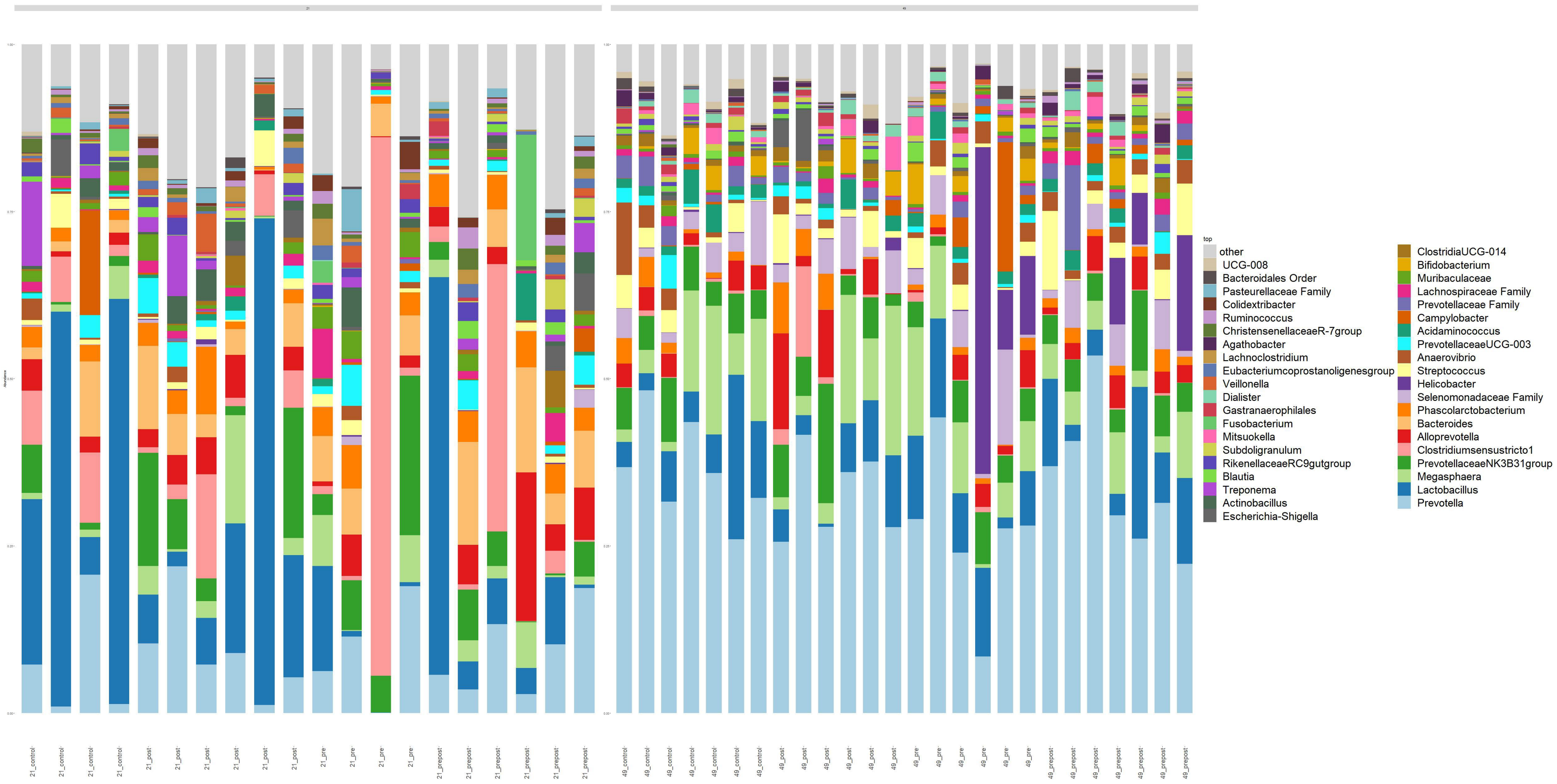

Figure S3. Stacked bar plot of bacterial taxon abundance within duodenum samples for all piglets sampled.

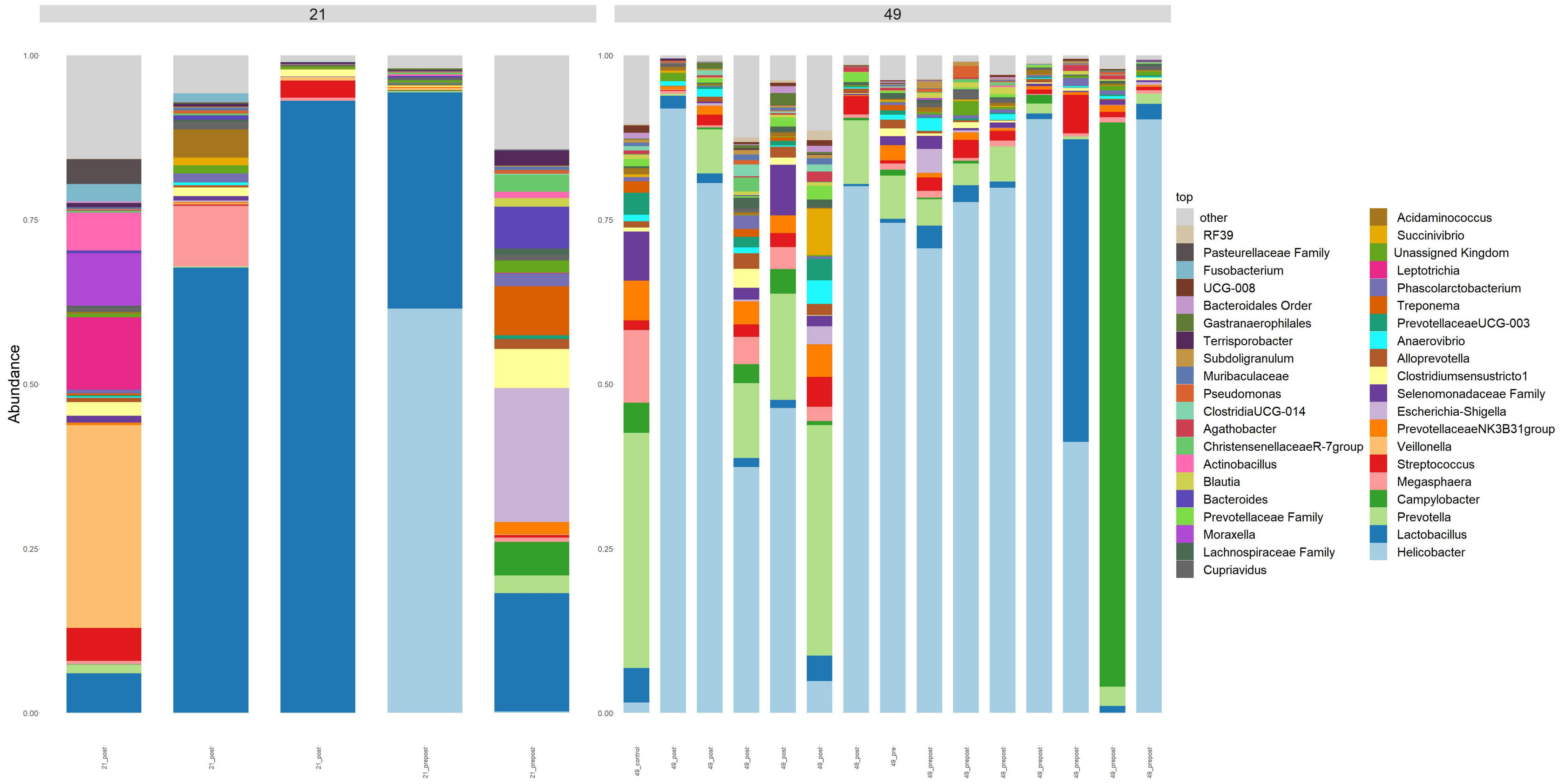

Figure S4. Stacked bar plot of bacterial taxon abundance within jejunum samples for all piglets sampled.

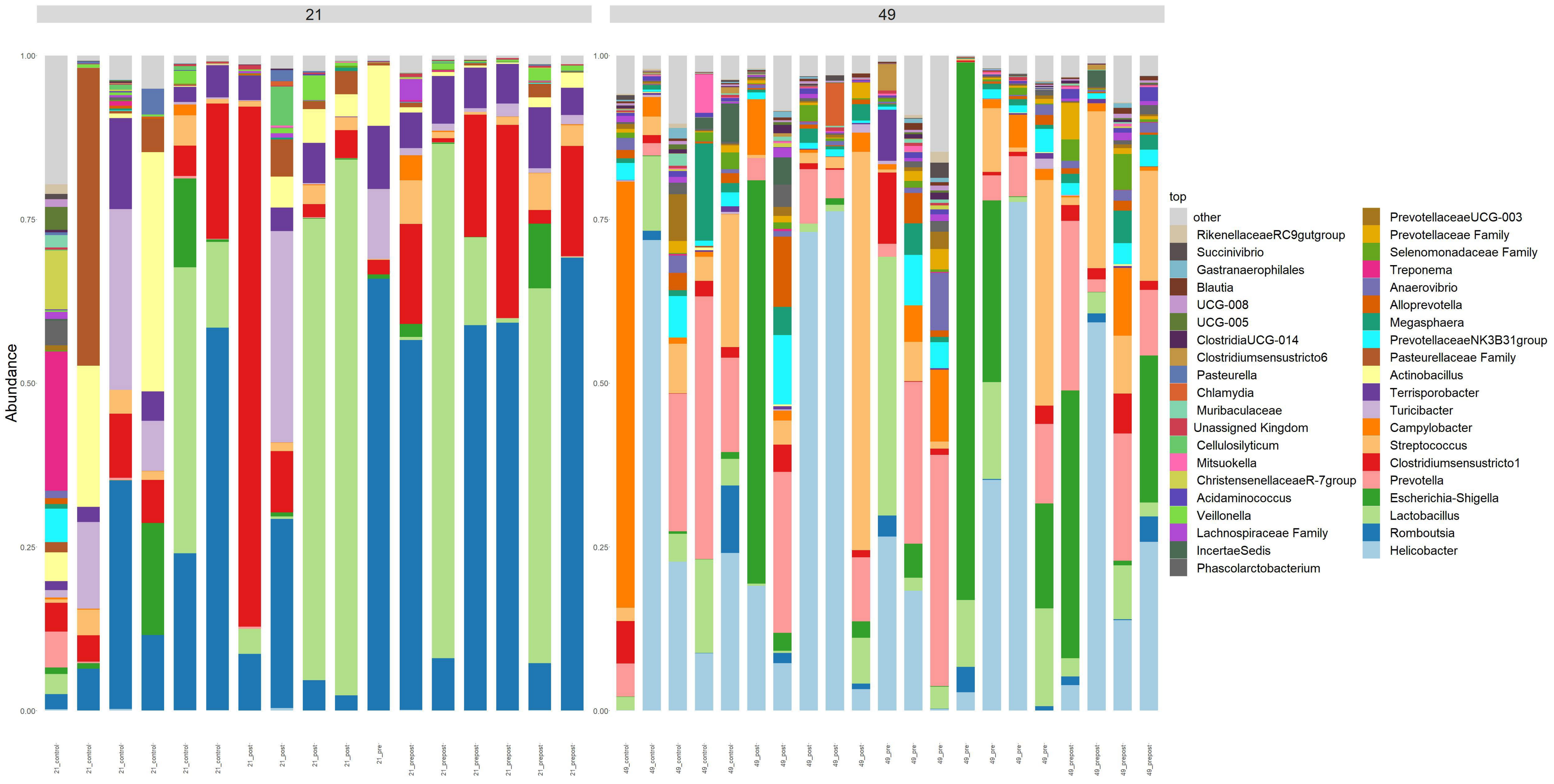

Figure S5. Stacked bar plot of bacterial taxon abundance within cecum samples for all piglets sampled.

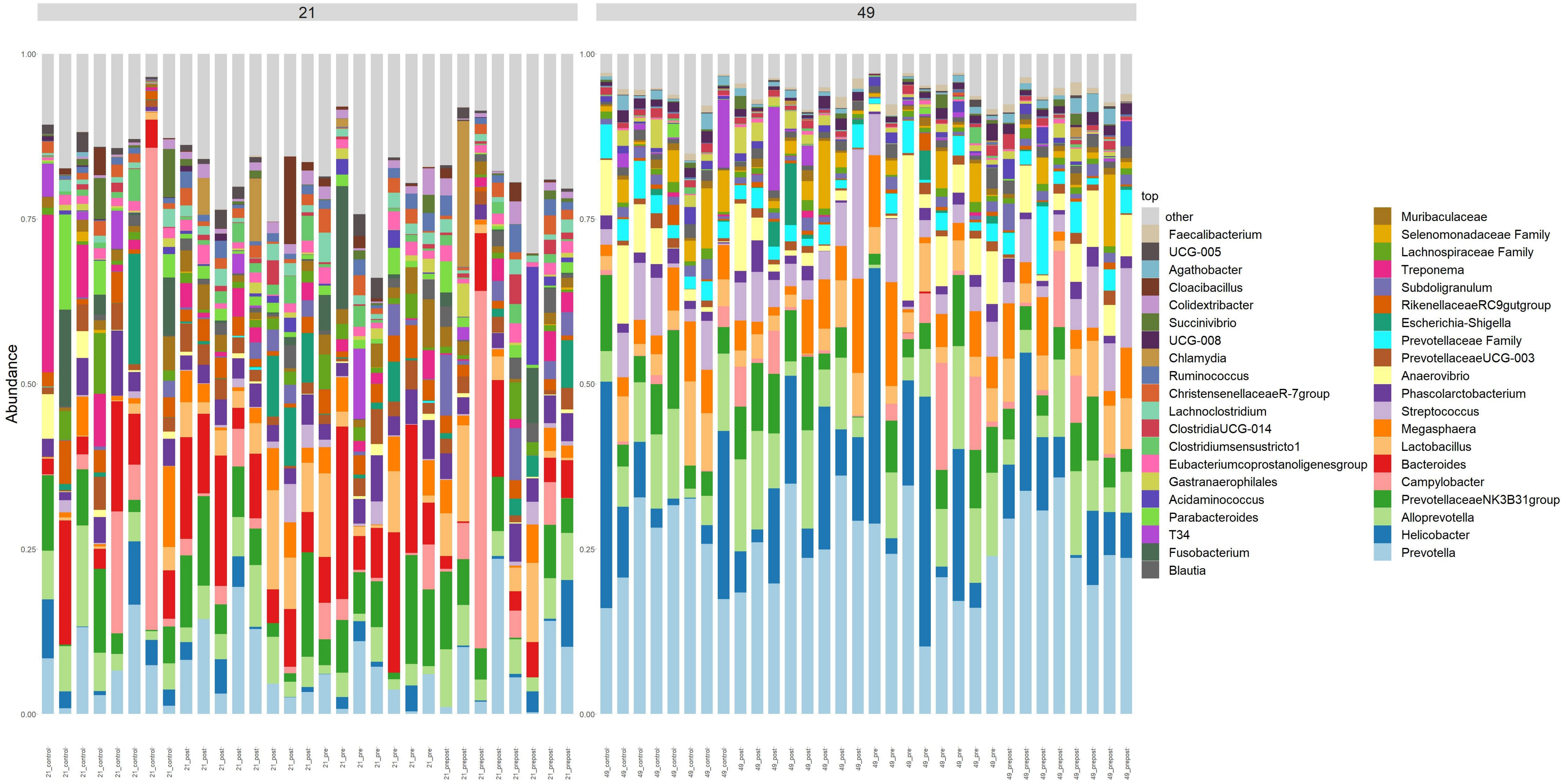

Figure S6. Stacked bar plot of bacterial taxon abundance within colon samples for all piglets sampled.

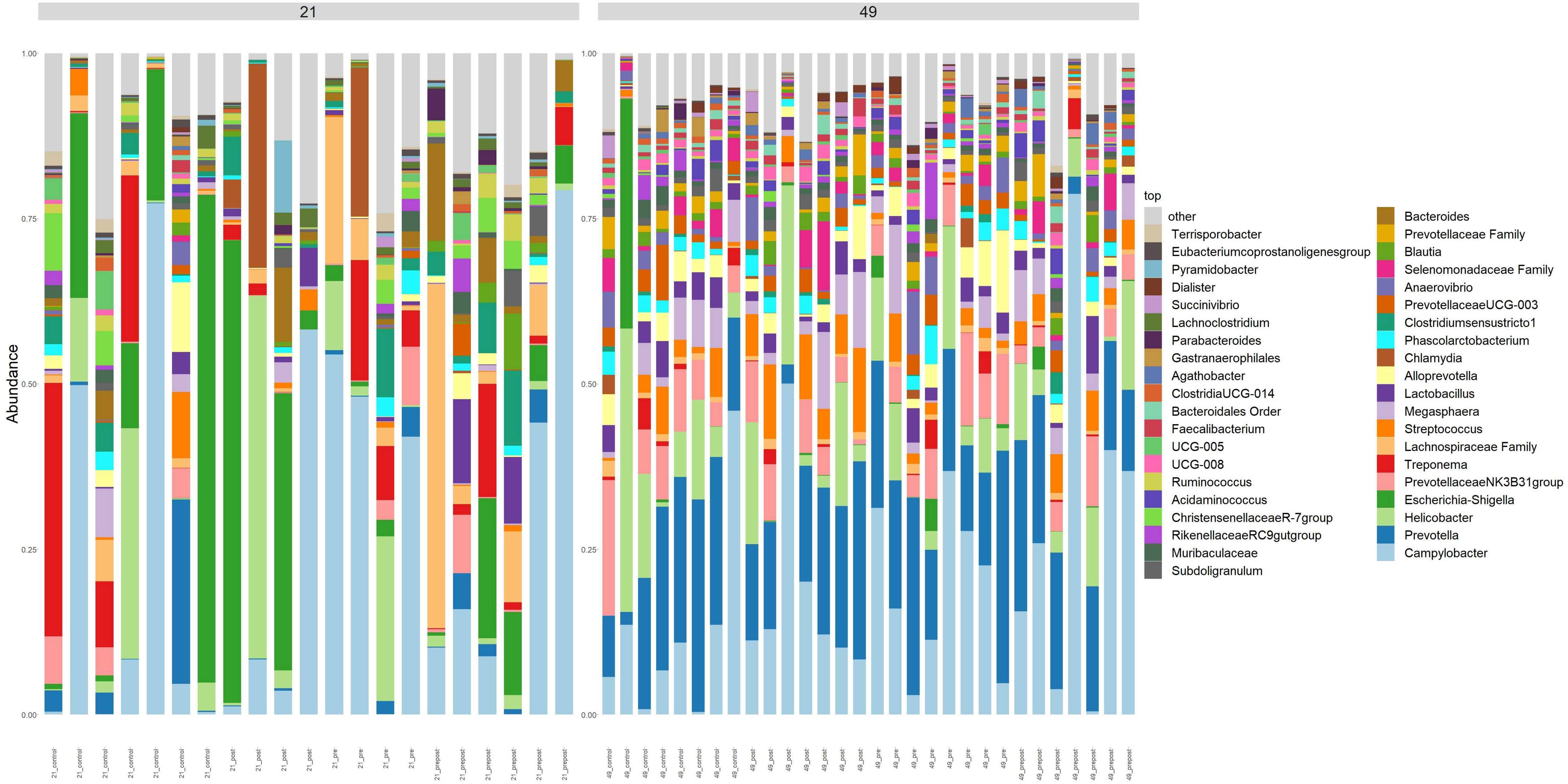

Figure S7. Stacked bar plot of fungal taxon abundance within stomach samples for all piglets sampled.

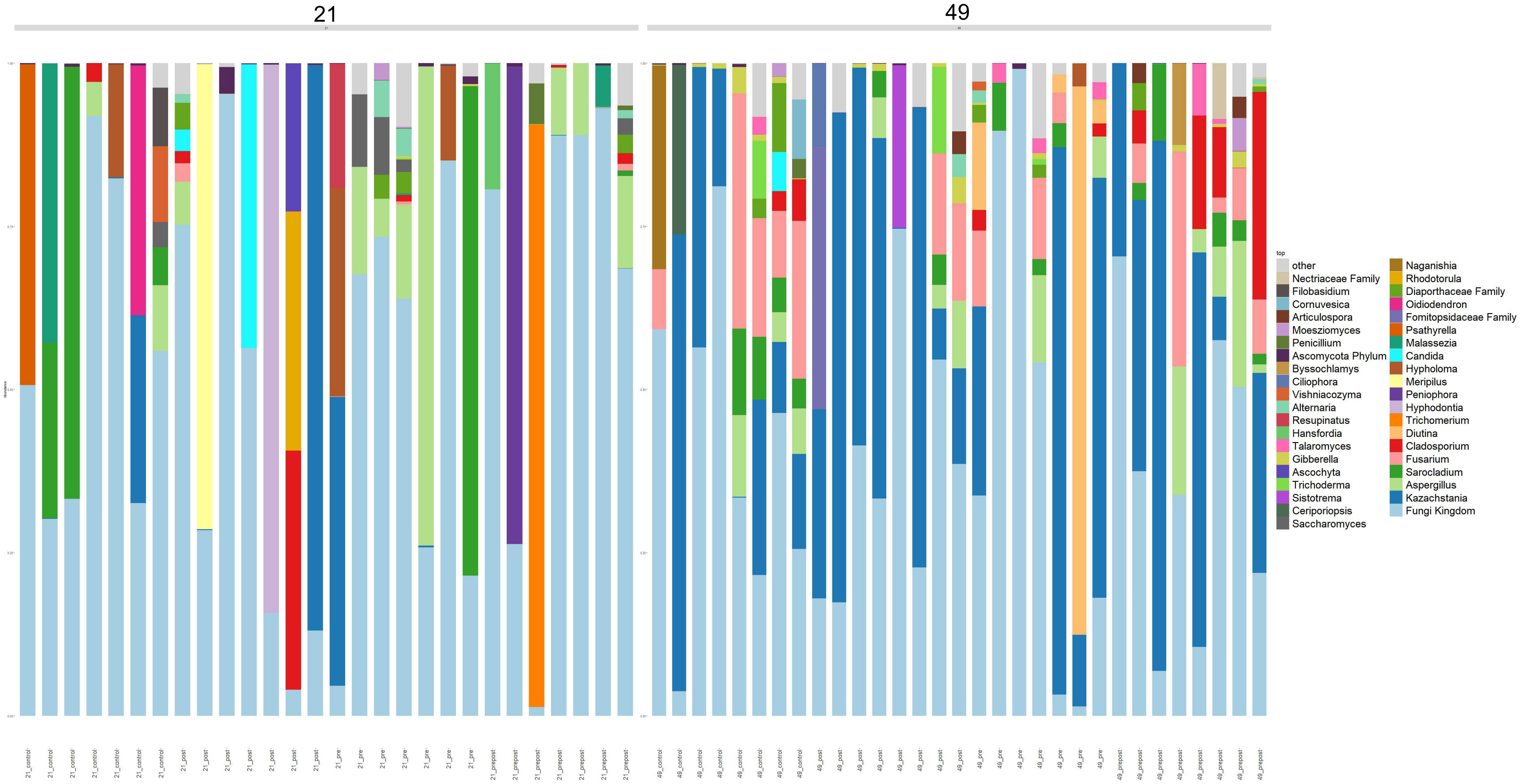

Figure S8. Stacked bar plot of fungal taxon abundance within duodenum samples for all piglets sampled.

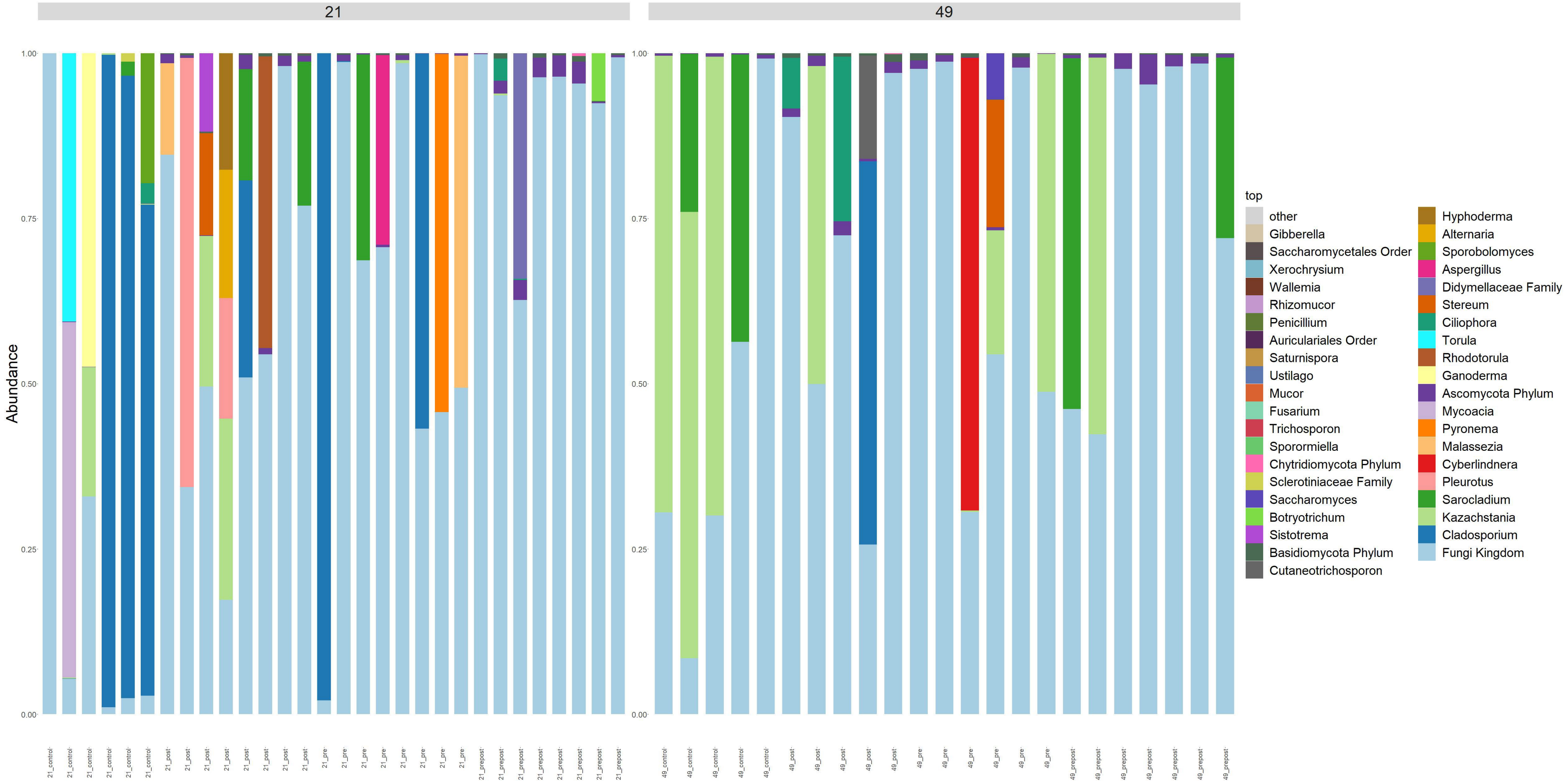

Figure S9. Stacked bar plot of fungal taxon abundance within jejunum samples for all piglets sampled.

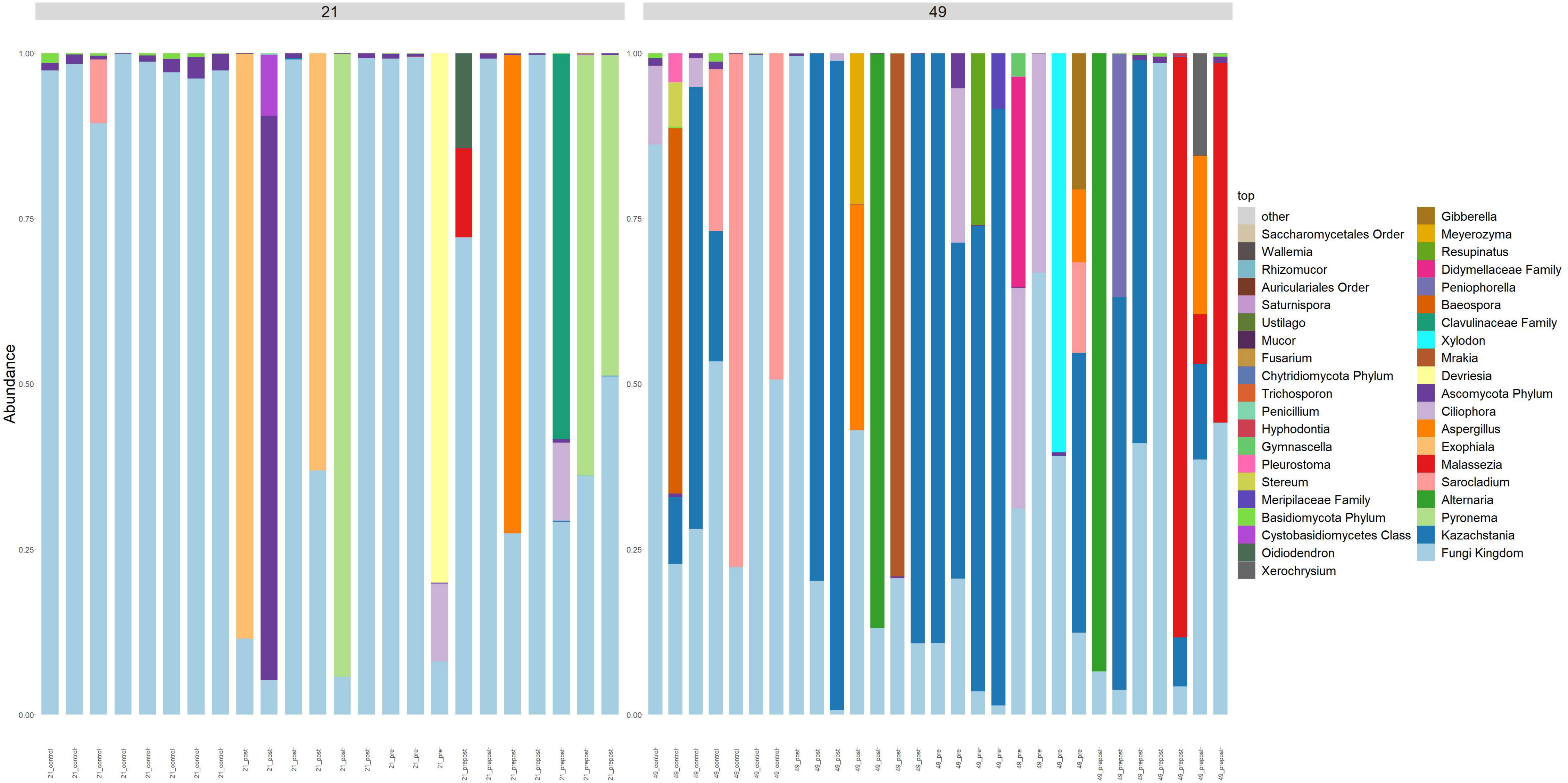

Figure S10. Stacked bar plot of fungal taxon abundance within cecum samples for all piglets sampled.

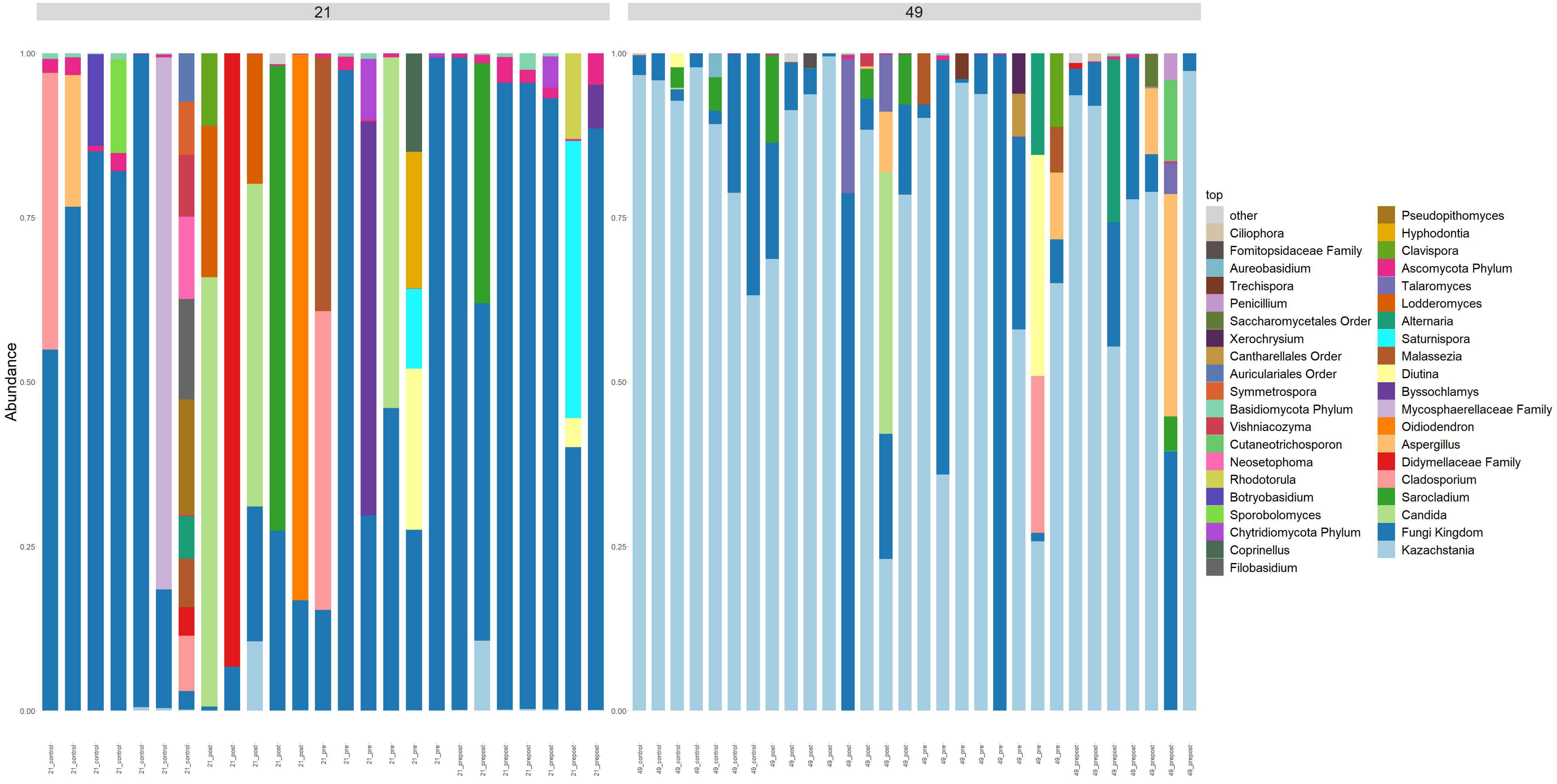

Figure S11. Stacked bar plot of fungal taxon abundance within colon samples for all piglets sampled.

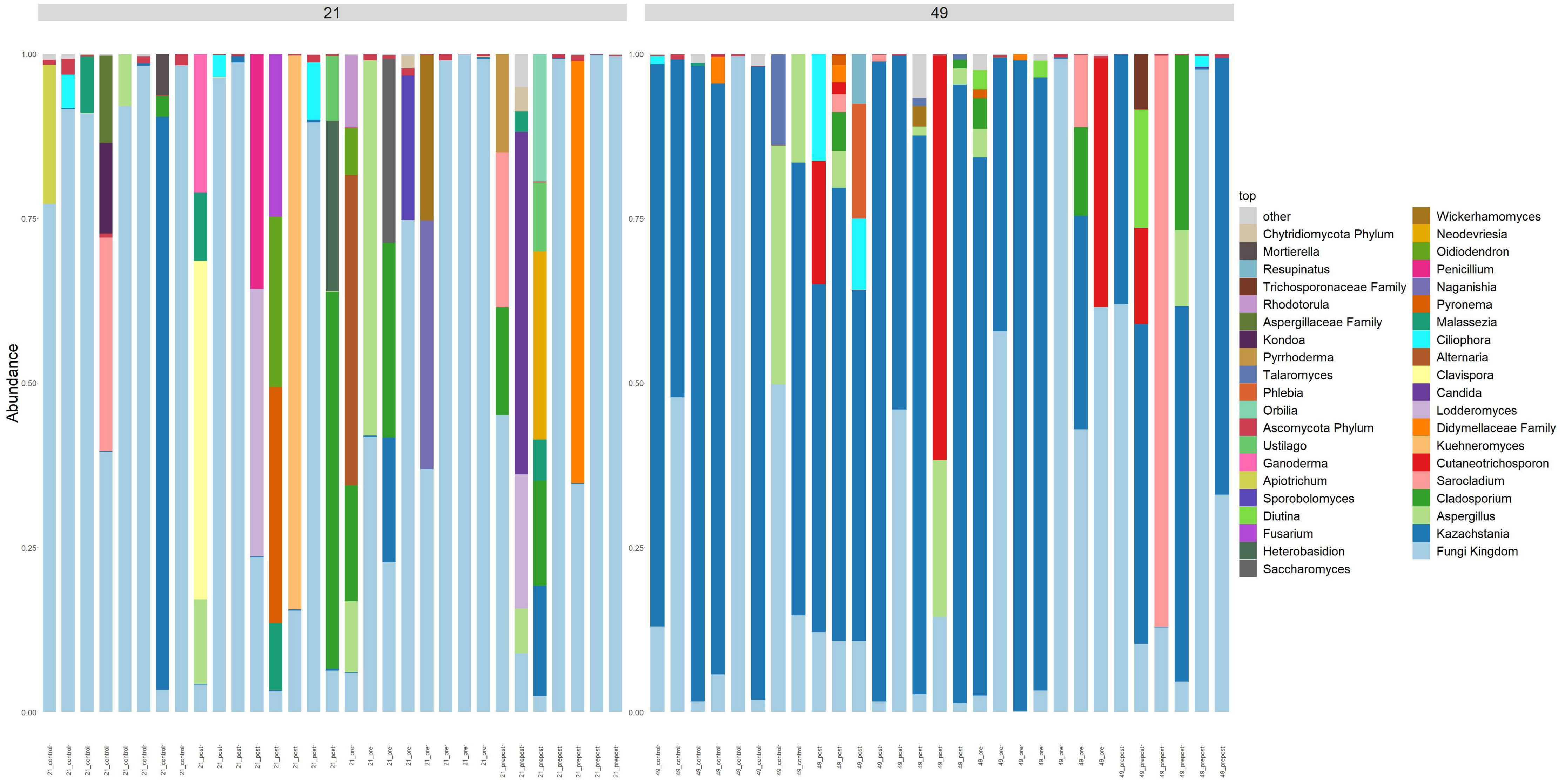

Figure S12. Stacked bar plot of bacterial taxon abundance within fecal samples for all piglets sampled.

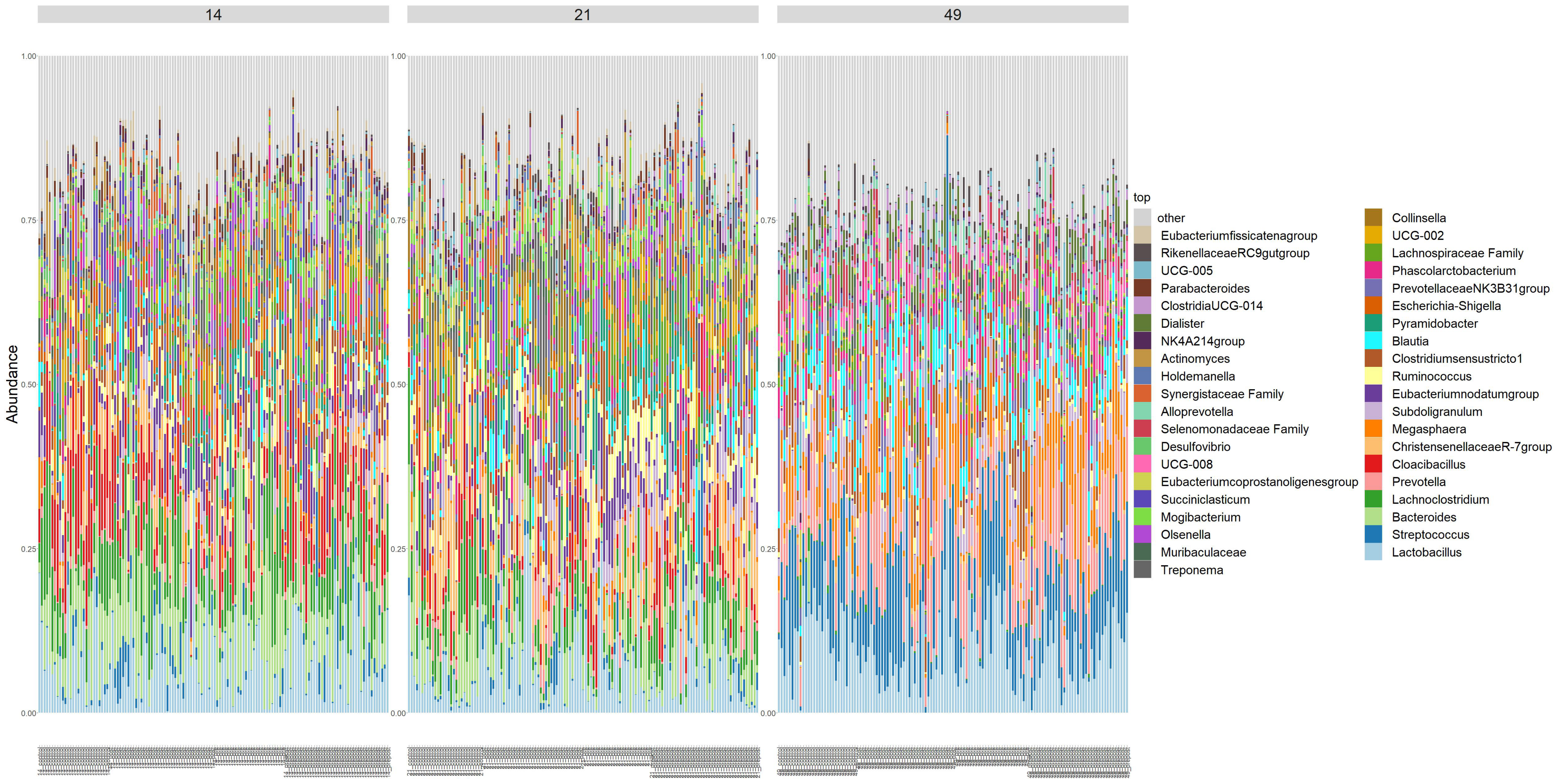

Figure S13. Stacked bar plot of fungal taxon abundance within fecal samples for all piglets sampled.

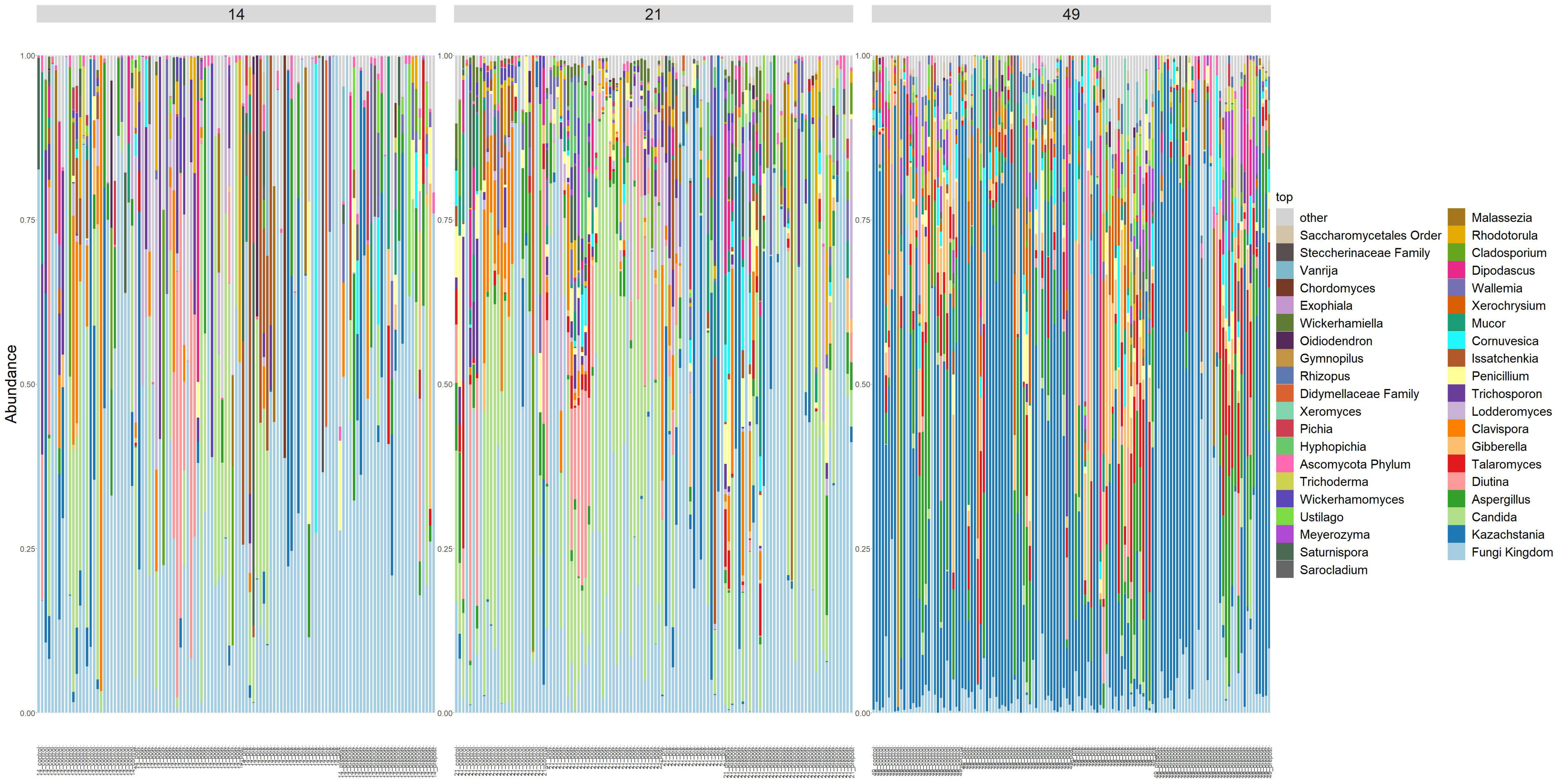

Figure S14. Relative *Kazachstania* abundance in feces by sow at each timepoint

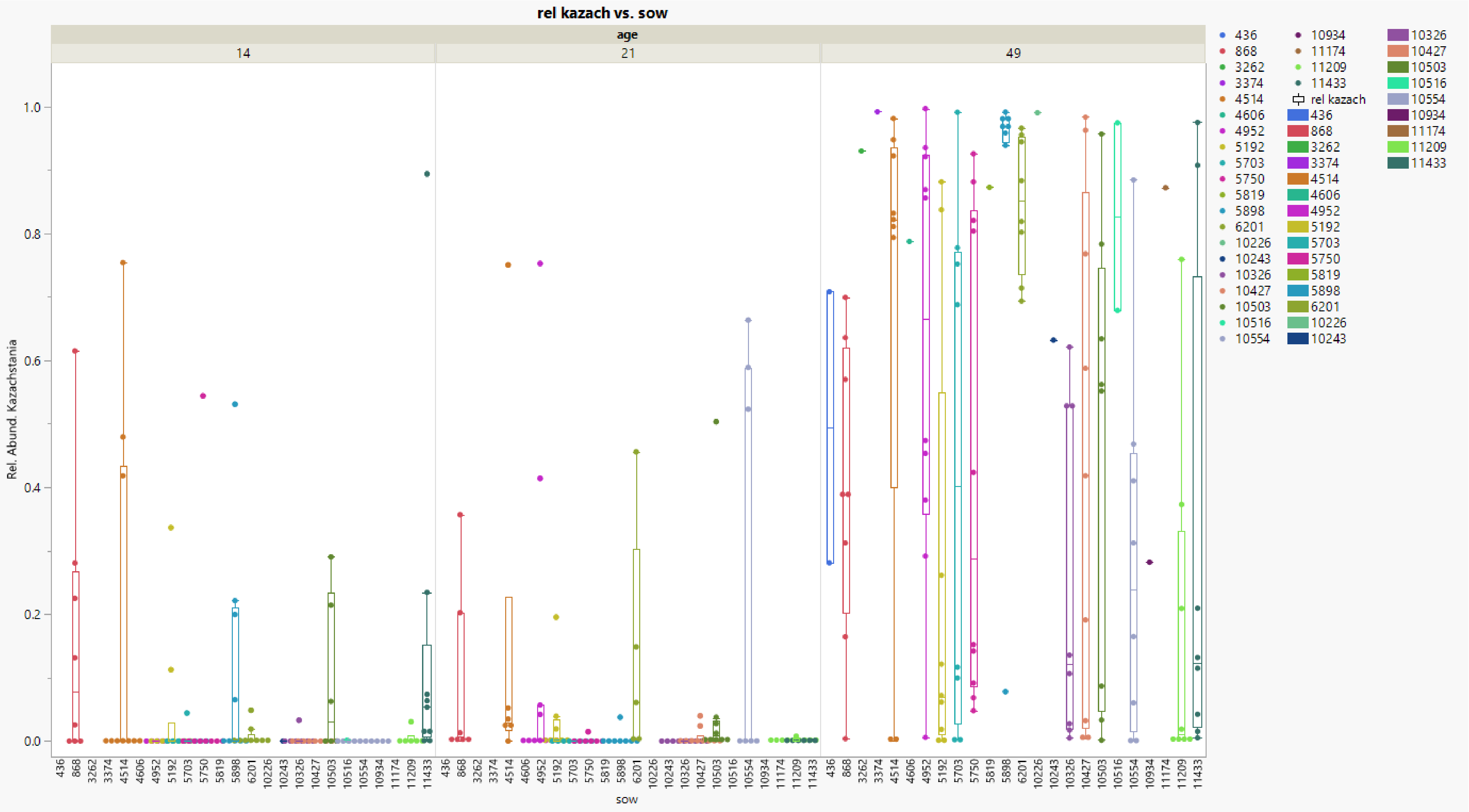

Supplement: Supplementary file 2 [file Data_Sheet_1.PDF]
